# *Piezo1* regulates cholesterol biosynthesis to influence neural stem cell fate during brain development

**DOI:** 10.1085/jgp.202213084

**Published:** 2022-09-07

**Authors:** Jamison L. Nourse, Vivian M. Leung, Hamid Abuwarda, Elizabeth L. Evans, Esmeralda Izquierdo-Ortiz, Alan T. Ly, Nguyen Truong, Samantha Smith, Harsh Bhavsar, Gabriella Bertaccini, Edwin S. Monuki, Mitradas M. Panicker, Medha M. Pathak

**Affiliations:** 1 Department of Physiology and Biophysics, University of California, Irvine, Irvine, CA; 2 Sue and Bill Gross Stem Cell Research Center, University of California, Irvine, Irvine, CA; 3 Center for Complex Biological Systems, University of California, Irvine, Irvine, CA; 4 Department of Biomedical Engineering, University of California, Irvine, Irvine, CA; 5 Department of Pathology and Laboratory Medicine, University of California, Irvine, Irvine, CA

## Abstract

Mechanical forces and tissue mechanics influence the morphology of the developing brain, but the underlying molecular mechanisms have been elusive. Here, we examine the role of mechanotransduction in brain development by focusing on *Piezo1*, a mechanically activated ion channel. We find that *Piezo1* deletion results in a thinner neuroepithelial layer, disrupts pseudostratification, and reduces neurogenesis in E10.5 mouse embryos. Proliferation and differentiation of *Piezo1* knockout (KO) mouse neural stem cells (NSCs) isolated from E10.5 embryos are reduced in vitro compared to littermate WT NSCs. Transcriptome analysis of E10.5 *Piezo1* KO brains reveals downregulation of the cholesterol biosynthesis superpathway, in which 16 genes, including *Hmgcr*, the gene encoding the rate-limiting enzyme of the cholesterol biosynthesis pathway, are downregulated by 1.5-fold or more. Consistent with this finding, membrane lipid composition is altered, and the cholesterol levels are reduced in *Piezo1* KO NSCs. Cholesterol supplementation of *Piezo1* KO NSCs partially rescues the phenotype in vitro. These findings demonstrate a role for *Piezo1* in the neurodevelopmental process that modulates the quantity, quality, and organization of cells by influencing cellular cholesterol metabolism. Our study establishes a direct link in NSCs between PIEZO1, intracellular cholesterol levels, and neural development.

## Teaser

PIEZO1, a mechanically gated ion channel, transduces mechanical cues in neural stem cells. Here, we show the impact of *Piezo1* knockout on neural development in vivo and find that cholesterol biosynthesis and lipid membrane composition are regulated by *Piezo1*.

## Introduction

Neural development involves the orchestration of a complex series of steps to generate the brain and spinal cord. While research has primarily focused on the influence of genetic and chemical factors on neural development, recent studies have demonstrated that mechanical forces also determine neural development and function (Abuwarda and Pathak, 2020). Mechanical cues encountered during development include changes in tissue stiffness, fluid shear flow, as well as hydrostatic forces from cerebrospinal fluid in the developing ventricles; however, the mechanisms that transduce mechanical signals in the developing brain are poorly understood. Potential molecular contributors to this process could include mechanically activated ion channels which can open rapidly in response to mechanical forces and mediate several downstream effects via electrochemical signals (Kefauver et al., 2020). The Piezo family of mechanically activated ion channels plays a role in mechanotransduction in many physiological systems, including vascular development (Li et al., 2014; Ranade et al., 2014), cardiovascular homeostasis (Rode et al., 2017), lymphatic development (Nonomura et al., 2018), red blood cell volume regulation (Cahalan et al., 2015), the baroreceptor response in neurons (Zeng et al., 2018), cartilage mechanics (Servin-Vences et al., 2017; Lee et al., 2014), bone formation (Sun et al., 2019), macrophage polarization responses (Atcha et al., 2021a), keratinocyte migration in wound healing (Holt et al., 2021), and neural stem cells (NSC) fate (Pathak et al., 2014).

We previously found that PIEZO1 channels are activated in response to both externally applied and cell-generated mechanical forces in human fetal brain-derived neural stem/progenitor cells (Pathak et al., 2014). Additionally, in vitro pharmacological inhibition or siRNA-mediated knockdown of PIEZO1 affected neural stem cell differentiation suggesting that PIEZO1 may function in embryonic brain development. Here, we sought to understand the role of PIEZO1 in early embryonic brain development by employing a constitutive *Piezo1* knockout (KO) mouse model (Ranade et al., 2014). We find that *Piezo1* null mutation results in aberrant neuroepithelial development and reduced proliferation and differentiation of NSCs. Using differential gene expression analysis, we find *Piezo1* KO brains have significantly reduced expression of genes in the cholesterol biosynthesis superpathway, which was corroborated by an altered lipid composition and lower levels of free cholesterol levels in *Piezo1* KO NSCs. Cholesterol supplementation of the NSC culture media partially rescued the differentiation phenotype in vitro. In summary, *Piezo1* is required for neural development through contributions to cholesterol metabolism.

## Materials and methods

### Animal maintenance

All experiments were carried out in accordance with approved Institutional Animal Care and Use Committee protocols and University of California, Irvine guidelines. *Piezo1* heterozygous null mice (*Piezo1*^∆/+^) were obtained from Jackson Laboratories (stock no. 026948). *Piezo1*^∆/+^ mice were bred with C57BL6/J to maintain the colony.

### Dissection and genotyping

Heterozygous mice were bred to generate a mixture of WT, heterozygous, and knockout embryos. Vaginal plug observation was considered embryonic day (E) 0.5*.* Dams were sacrificed at specified time points, and embryos were harvested. Each embryo was genotyped through a commercial vendor (Transnetyx).

### Hematoxylin & eosin (H&E) staining

E10.5 embryos were drop-fixed immediately after harvesting in Bouin’s fixative (#26386-01; EMS) for 24 h at 4°C. Fixed embryos were paraffin-embedded, sectioned sagittally at a thickness of 5 µm, and stained with H&E using standard procedures.

### In situ hybridization (ISH) and immunofluorescence of tissue slices

E10.5 embryos were drop-fixed in 4% v/v paraformaldehyde (PFA; #15710; EMS) overnight at 4°C. We found that E10.5 *Piezo1* KO embryonic brain tissue is considerably more fragile than WT, requiring paraffin embedding to maintain tissue structures during processing. Fixed embryos were paraffin-embedded and sectioned sagittally at 5 µm thickness. Sections were deparaffinized with xylene and 100% ethanol and incubated in RNAscope Target Retrieval Reagent (#322000; ACD Bio) for 15 min at 95°C. ISH was performed using the RNAscope Multiplex Fluorescent Reagent Kit v2 (#323100; ACD Bio) using the manufacturer’s protocol for formalin-fixed paraffin-embedded (FFPE) sections. WT and *Piezo1* knockout tissue sections were incubated with a *Piezo1* Probe (#400181; ACD Bio). Sections were then immunolabeled with anti-TUJ1 antibody (#2G10; Santa Cruz) to identify early neuronal populations; in separate experiments PFA-fixed, paraffin-embedded sections were also immunolabeled with anti-Nestin (#AB134017; Abcam) and anti-Sox2 (#AB5603; Millipore) antibodies. For immunofluorescence of tissue slices after deparaffinization, sections were rehydrated in a series of graded ethanol from 95, 80, to 60% for 5 min each at room temperature, washed with water twice for 1 min, boiled in IHC Select Citrate Buffer pH 6 (149550; EMD) for 20 min, permeabilized with 0.3% Triton X-100 for 15 min at room temperature, blocked with 5% BSA; (#001-000-162; Jackson ImmunoResearch) for 1 h at room temperature, and incubated with primary antibodies overnight at 4°C. The following day, sections were incubated with secondary antibodies for 1 h at room temperature. Nuclei were labeled with 6 µg/ml Hoechst (#H1399; Thermo Fisher Scientific) for 5 min at room temperature and mounted with Prolong Diamond (#P36965; Thermo Fisher Scientific). See Table S3 for antibodies used.

### Whole-mount immunolabeling

#### Pretreatment with methanol

Embryos were drop-fixed in 4% v/v PFA (#15710; EMS) overnight at 4°C*.* Fixed samples were dehydrated in methanol (#A412-4; Thermo Fisher Scientific)/H_2_O series of 20, 40, 60, 80, and 100%; 1 h each at room temperature. Samples were then incubated overnight in two parts dichloromethane (#270997; Sigma-Aldrich)/one part methanol with shaking at room temperature. The following day, samples were washed twice with 100% methanol and then chilled for 1 h at 4°C. Samples were bleached in chilled fresh 5% H_2_O_2_ (#H325; Thermo Fisher Scientific) in methanol (1 volume 30% H_2_O_2_ to 5 volumes MeOH) overnight at 4°C. Samples were rehydrated the next day with methanol/H_2_O series of 80, 60, 40, 20%, and phosphate-buffered saline (PBS), 1 h each at room temperature. Finally, samples were washed in PBS/0.2% Triton X-100 (#11332481001; Sigma-Aldrich) for 1 h twice.

#### Immunolabeling

Pretreated samples were incubated in PBS/0.2% Triton X-100/20% dimethylsulfoxide (DMSO) (#276855; Sigma-Aldrich)/0.3 M glycine (#G7126; Sigma-Aldrich) at 37°C overnight, then blocked in PBS/0.2% Triton X-100/10% DMSO/6% donkey serum (#S30; EMD Millipore) at 37°C for 1 d. Samples were washed in PBS/0.2% Tween-20 (#P1370; Sigma-Aldrich) with 10 μg/ml heparin (#H3149; Sigma-Aldrich; PTwH) for 1 h twice, then incubated in primary antibody dilutions (see Table S3) in PTwH/5% DMSO/3% donkey serum at 37°C for 2 d. After primary antibody incubation, samples were washed in PTwH four to five times until the next day. Samples were then incubated with secondary antibodies (see Table S3) in PTwH/3% Donkey Serum for 1 d at 37°C. After incubation, samples were washed in PTwH four to five times until the following day. Following this, embryos were embedded in 2% low melting agarose before clearing.

#### Tissue clearing

Immunolabeled samples were cleared using the iDisco protocol (Renier et al., 2014). Immunolabeled samples were dehydrated using methanol/H_2_O series: 20, 40, 60, 80, and 100%; 1 h each at room temperature. Samples were then left fully submerged in 100% methanol overnight, before being incubated for 3 h with shaking in two parts dichloromethane/one part methanol at room temperature. Samples were then washed twice in 100% methanol for 15 min each before being incubated in dibenzyl ether (#108014; Sigma-Aldrich) overnight before imaging.

### Bulk RNA sequencing and analysis

The brains of E10.5 embryos were dissected, flash-frozen, and stored at −80°C until RNA was harvested with the total RNA isolation kit (#732-6820; Bio-Rad). Total RNA was monitored for quality using the Agilent Bioanalyzer Nano RNA chip and Nanodrop absorbance ratios for 260/280 nm and 260/230 nm and RNA Integrity Number (RIN) scores of 9.5–10. Library construction, sequencing, and post-processing of the run to generate the FASTQ files were performed at the UCI Genomics High Throughput Facility. Library construction was performed according to the Illumina TruSeq mRNA stranded protocol, and mRNA was enriched using oligo dT magnetic beads. The enriched mRNA was chemically fragmented. First-strand synthesis used random primers and reverse transcriptase to make cDNA. After second strand synthesis, the double-stranded cDNA was cleaned using AMPure XP beads and the cDNA was end-repaired and then the 3′ ends were adenylated. Illumina barcoded adapters were ligated on the ends, and the adapter-ligated fragments were enriched by nine cycles of PCR. The resulting libraries were validated by qPCR and sized by Agilent Bioanalyzer DNA high sensitivity chip. The multiplexed libraries were sequenced using paired-end 150 cycles chemistry on NovaSeq 6000. Raw reads were trimmed using a quality threshold of 15 and Truseq adapter sequence, trimmed reads shorter than 20 bp were discarded. Reference genome sequence mm10 and its transcriptome annotation were collected and indexed for mapping. Gene expression level was quantified using featureCount for raw counts. Statistical comparison was made using DESeq2 to obtain differential gene expression data (Love et al., 2014). Principal component analysis (PCA) was conducted in R (R Core Team, 2022) using the package DESeq2 and function plotPCA with default settings. Differential gene expression data were analyzed with Ingenuity Pathway Software (QIAGEN Inc., https://digitalinsights.qiagen.com/IPA) with the algorithms developed for use in QIAGEN IPA (Krämer et al., 2014) using only genes differentially expressed by 1.5-fold L2R [±0.6] and with the false discovery rate (pAdj) set at pAdj<0.0001 with no restrictions on the Ingenuity library of datasets.

### Mouse neural stem cell culture

#### Isolation and expansion

Neural stem cell cultures were isolated from E10.5 brains. For neurosphere cultures, cells were plated onto non-adherent cultureware at 100 K/ml of media. All mouse NSC cultures were maintained in proliferation media: Dulbecco’s modified Eagle’s media (#11995-065; Thermo Fisher Scientific), supplemented with 1× N2 (#17502048; Thermo Fisher Scientific), 1× B27 (#17504044; Thermo Fisher Scientific), 1 mM sodium pyruvate (#11360070; Thermo Fisher Scientific), 2 mM Glutamax (#35050061; Thermo Fisher Scientific), 1 mM N-acetylcysteine (#A7250; Millipore Sigma), 10 ng/ml b-FGF (#100-18B; Peprotech), 20 ng/ml EGF (#AF-100-15; Peprotech), and 2 µg/ml heparin (#H3149; Millipore Sigma). To passage neurospheres, cultures were dissociated using the Neurocult Chemical Dissociation Kit (#05707; Stem Cell Technologies). For staining and differentiation experiments, neurospheres were dissociated and cells were seeded at a density of 30 K/cm^2^ onto #1.5 acid-washed coverslips coated with laminin (10 µg/ml).

#### Immunofluorescence

Cells were fixed in 4% v/v PFA, 5 mM MgCl_2_, 10 mM EGTA, and 40 mg/ml sucrose in PBS for 10 min and permeabilized with 0.3% Triton X-100 in PBS for 5 min, blocked with 5% BSA in PBS for 1 h at room temperature, and incubated with primary antibodies in 1% BSA overnight at 4°C. The following day, cells were incubated with secondary antibodies for 1 h at room temperature. See Table S3 for antibodies used. Nuclei were labeled with 6 µg/ml Hoechst-33342 (#H1399; Life Technologies) in PBS for 5 min at room temperature, and samples were mounted using Prolong Diamond (#P36965; Thermo Fisher Scientific).

#### Filipin III and Nile Red cell staining

Adherent mNSC cells plated on 35 mm glass bottom dishes (#D35-20-1.5-N; Cellvis) were fixed with 4% PFA for 10 min and washed three times with PBS. Fixed cells were stained with 1 µg/ml Nile Red (#N1142; Invitrogen) or 50 µg/ml Filipin III (#F4767; Sigma-Aldrich). Filipin III was reconstituted with anhydrous DMSO (#276855; Sigma-Aldrich) at 2 mg/ml and used either the same day or within 2 wk (stored immediately after DMSO reconstitution at −80°C). Since Filipin III is very unstable and bleaches rapidly, cells were stained one sample at a time with Filipin III freshly diluted to 50 µg/ml in PBS containing 500 nM nuclear counterstain siR-DNA (#CY-SC007; Cytoskeleton, Inc.) for 45 min in the dark at room temperature. Cells were washed once with 1.5 ml PBS and imaged immediately. Alternatively, fixed cells were stained with Nile Red (#N1142; Thermo Fisher Scientific) for 20 min at 37°C in PBS, nuclei counterstained with 6 μg/ml Hoechst-33342 (#H1399; Thermo Fisher Scientific), washed three times with PBS, and imaged in PBS.

#### Differentiation assay

Neurospheres were dissociated and cells were seeded at a density of 30 K/cm^2^ onto #1.5 acid-washed coverslips coated with laminin (10 µg/ml). After 24 h, media was removed and differentiation media was added (same composition as the proliferation media, but without bFGF, EGF, and heparin). Cells were fed every 2 d and fixed after 4 d or fixed after 10 d in differentiation media. For cholesterol rescue experiments, cholesterol–methyl-β-cyclodextrin (#C4951, Lot #SLCB4694; Sigma-Aldrich) at 10 µg/ml (equivalent to 1.3 µM cholesterol) was added to the media at the time of plating and during media changes. Antibodies used for identifying differentiated cell types include astrocytic marker glial fibrillary acidic protein (GFAP), neuronal marker microtubule associated protein (MAP2), and oligodendrocyte marker (O4). GFAP staining of astrocytes is distinguished by a high GFAP signal compared to very low levels found in proliferating NSCs. See Table S3 for antibodies used.

#### Proliferation assay

Neurospheres were dissociated and seeded in triplicate for each biological replicate (isolated from individual embryos) in proliferation media at a density of 10,000 cells/well in a 96-well plate (#CC7682-7596; USA Scientific) coated with laminin (10 µg/ml) and allowed to recover for 18–22 h. To visualize nuclei, 100 µl proliferation media containing 250 nM siR-DNA Kit (#CY-SC007; Cytoskeleton, Inc.) was added per well. Cerivastatin (#SML0005; Sigma-Aldrich) was added at 0, 20, 50, and 100 nM. Live cells were imaged with a 10× phase objective with both phase contrast and fluorescence (using filter cube Ex 565–605 nm/Em 625–705 nm) at four to five positions per well every 2 h in the IncuCyte S3 Live-Cell Analysis System for 60 h. Fold proliferation was determined by analysis with Sartorius software of the number of nuclei at each time point in each image and normalized to the number of nuclei at time point 0 for each image. Data presented are from three wells for each embryo. A minimum of three embryos for WT and for KO were used per experiment.

### Flow cytometry

One million single cells from dissociated neurospheres were stained with 1 µg/ml Nile Red for 20 min at 37°C in proliferation media. Cells were pelleted at 110 × *g* for 5 min and washed three times with PBS. Zombie Violet fixable viability kit (#423113; BioLegend) was added at 1:2,000 dilution in PBS. A total of 10,000–30,000 events were analyzed with a BD Fortessa X20 flow cytometer using a 488 nm laser with a 505 nm long pass filter (green) and a 561 nm laser with a 600 nm long pass filter (red). FlowJo v10.7 Software (BD Life Sciences) calculated the geometric mean of green (488 nm signal) and red (561 nm signal) from cells and the ratio of green to red signal was assessed.

### Imaging

H&E-stained embryos were imaged with Leica Aperio VERSA 200 (40× objective). ISH and immunofluorescence-stained sections were imaged with Olympus FV3000S laser-scanning confocal microscope (40× [NA 1.25] and 60× [NA 1.25] objectives). Whole-mount–stained embryos were imaged by Zeiss Z-1 Lightsheet Microscope (5× [NA 0.16] objective). Immunolabeled embryo sections and cultured cells were imaged with Keyence BZ-X810 Widefield Microscope (10× [NA 0.45], 20× [NA 0.75], and 40× [NA 0.95] objectives). Filipin-labeled cultured cells were imaged with Keyence BZ-X810 Widefield Microscope (60× [NA 1.4] oil objective). The proliferation of NSCs was imaged with Incucyte S3 Live Cell Imaging system (10× [NA 0.3] objective) and analyzed with S3 2020A GUI software. The Incucyte system uses a 12-bit CMOS camera (scientific grade CMOS camera) and contrast-based autofocus to acquire images. Nile Red stained cells were imaged with Zeiss Elyra 7 for Lattice SIM Microscopy with 405, 488, and 561 nm lasers, PlanApo 63× oil immersion objective (NA 1.40), and acquired using a dual camera: 2 pco.edge 4.2 camera system with lattice sim leap mode. Subsequently, Nile Red images were processed using Zeiss’s automated sim leap mode pipeline. See Table S4 for microscope details.

### Analysis

For Figs. 2 C; 3, B and C; 4, D and F; 5, A and B; S7; and S8, Gardner-Altman estimation plots were generated, Cohen’s *d* was calculated, and P values were calculated by two-sided permutation t-test (except for Figs. 2 C and 4 F) using an online estimation stats tool (www.estimationstats.com; Ho et al., 2019). The estimation stats tool shows both groups plotted on the left axes; the mean difference (depicted as a large black dot) is plotted on a floating axis on the right as a bootstrap sampling distribution with 5,000 bootstrap samples taken; the 95% confidence interval was bias-corrected and accelerated and is indicated by the ends of the vertical error bar. For each permutation P value, 5,000 reshuffles of the control and test labels were performed; P value(s) reported are the likelihood(s) of observing the effect size(s), if the null hypothesis of zero difference is true. OriginPro 2022 (OriginLab Corporation) was used for calculating P values for Figs. 2 C, 3 E, 4 F, and S5 B using two-sample t-test and for generating the plot used in Fig. 3 E. TUJ1 area was determined by calculating the percent area occupied by TUJ1 signal using FIJI (Schindelin et al., 2012). GFAP, MAP2, and O4 positive cells were manually counted with the FIJI plug-in (Analysis → Cell Counter). Filipin signal was measured in FIJI using the sum of the values of the pixels in the image (RawIntDens) after background subtraction of the mean of five cell-free regions of each image. Nuclei were counted in each image and the Filipin signal per cell was obtained by dividing the RawIntDens by the number of nuclei in each image.

### Online supplemental material

**Figure S1.**
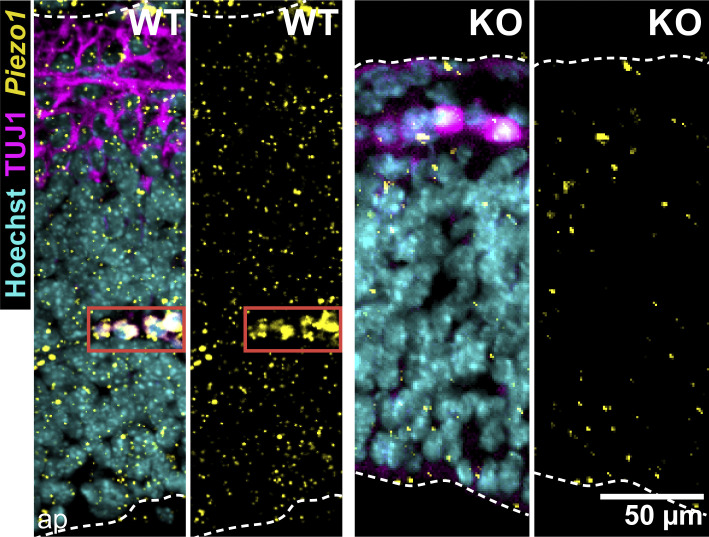
**Fluorescent ISH of *Piezo1* mRNA in the E10.5 mouse hindbrain.** Representative ISH images of the hindbrain region of WT and *Piezo1* KO mutant embryo sections. *Piezo1* mRNA shown in yellow, TUJ1 antibody staining of neurons shown in magenta, and Hoechst-stained nuclei in cyan. The white dashed lines demarcate the neuroepithelium, and the red solid box highlights a blood vessel; ap, apical/ventricular border. Images are representative of *n* = 3 embryos for WT and *n* = 2 embryos for KO. WT panels are duplicated from Fig. 2 A for reference. Related to Fig. 2 A.

**Figure S2.**
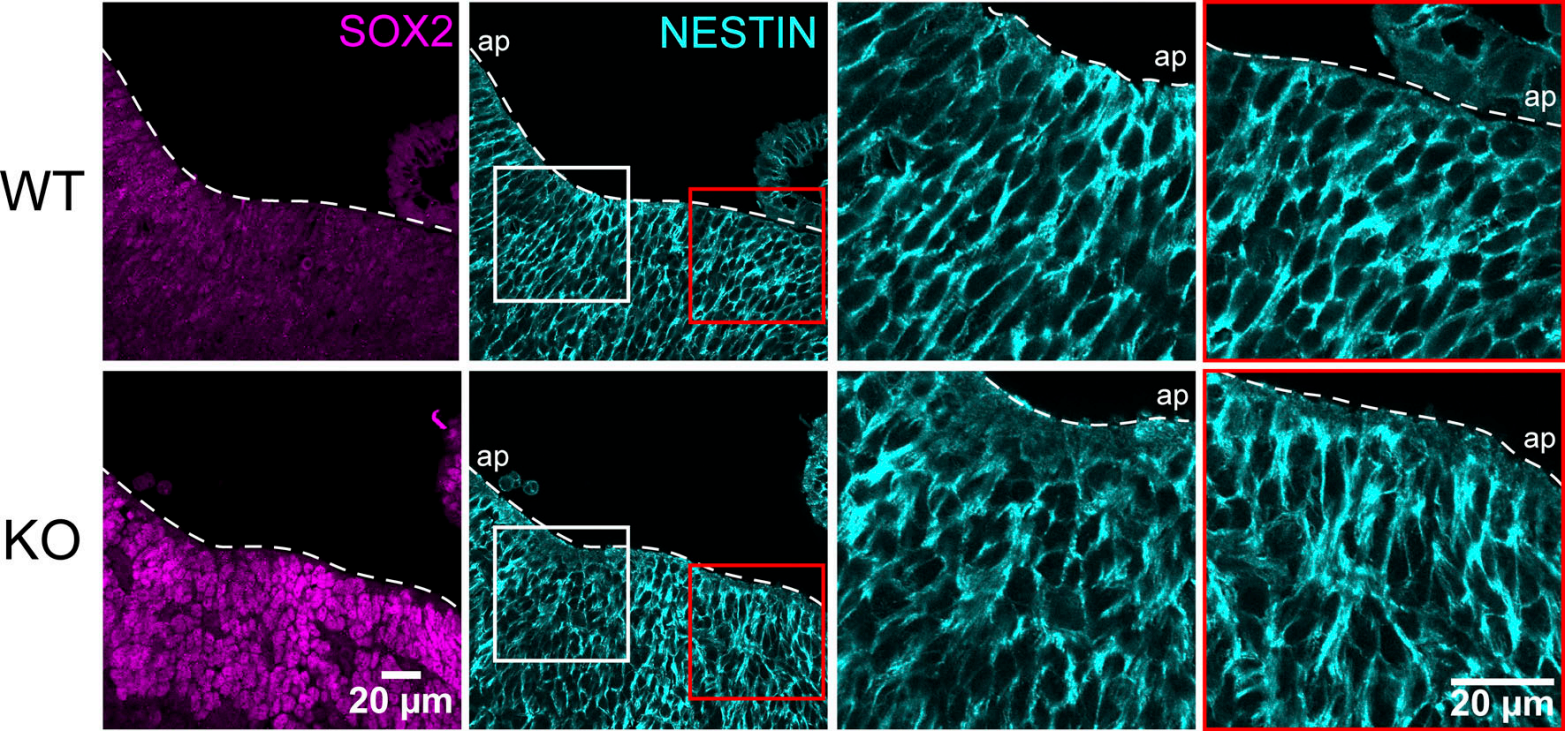
***Piezo1* KO results in a disorganized neuroepithelium in vivo.** Representative WT and *Piezo1* KO images of the hindbrain region from embryo sections immunostained with SOX2 (magenta) and NESTIN (cyan). The white dashed lines demarcate the neuroepithelial borders; ap, apical/ventricular border. White and red boxed regions in the NESTIN panel are shown at higher magnification on the right. Note the pseudostratified organization of cells along the apical–basal axis in the WT sample, which is disrupted in the *Piezo1* KO. Data are representative from *n* = 3 embryos for WT and *n* = 3 embryos for *Piezo1* KO from two independent experiments. Related to Fig. 2 B.

**Figure S3.**
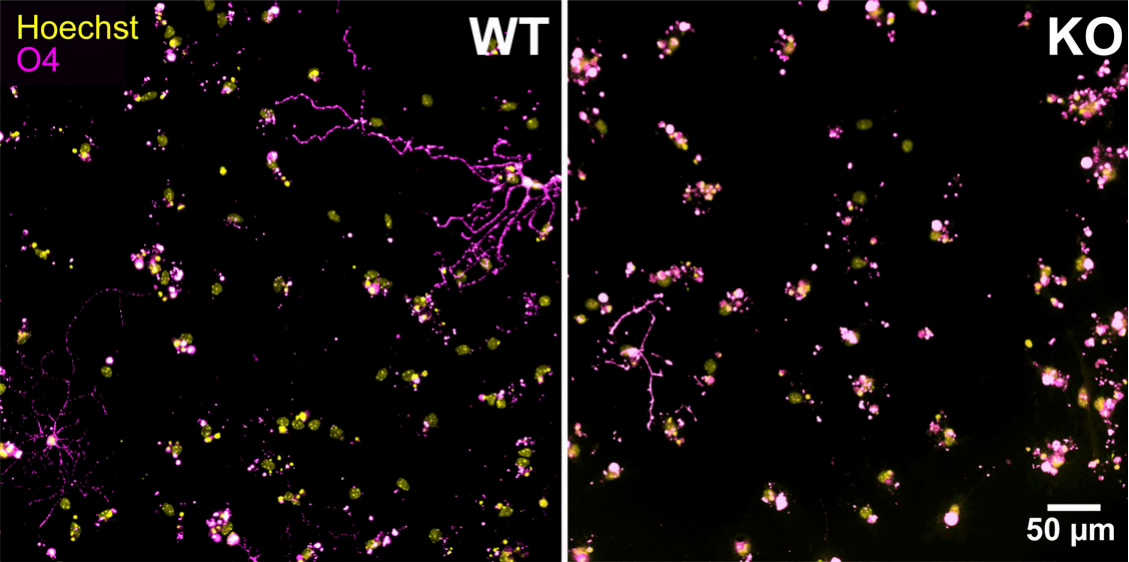
**Oligodendrocyte differentiation is reduced in *Piezo1* KO NSCs.** Representative fluorescence images of E10.5 WT and *Piezo1* KO NSCs differentiated for 10 d in vitro and immunostained for the Oligodendrocyte marker, O4 (magenta) and counterstained nuclei with Hoechst (yellow). *n* = 6 embryos for WT and *n* = 4 embryos for *Piezo1* KO from two experiments. Related to Fig. 3 C.

**Figure S4.**
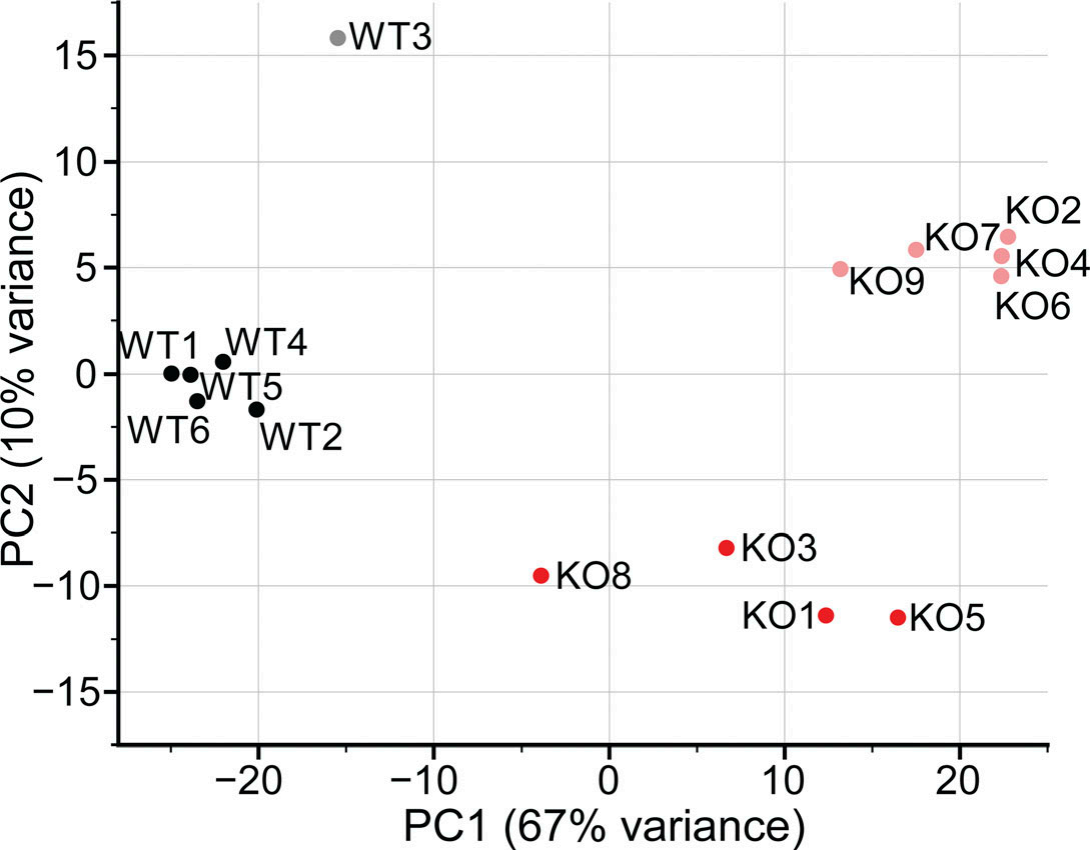
**PCA plot of 15 WT and *Piezo1* KO RNA-seq samples.** Numbers indicate embryo name; black circles, WT female embryos; gray circles, WT male embryos; red circles, *Piezo1* KO females; light red circles, *Piezo1* KO males. Related to Fig. 4 A and Table S1.

**Figure S5.**
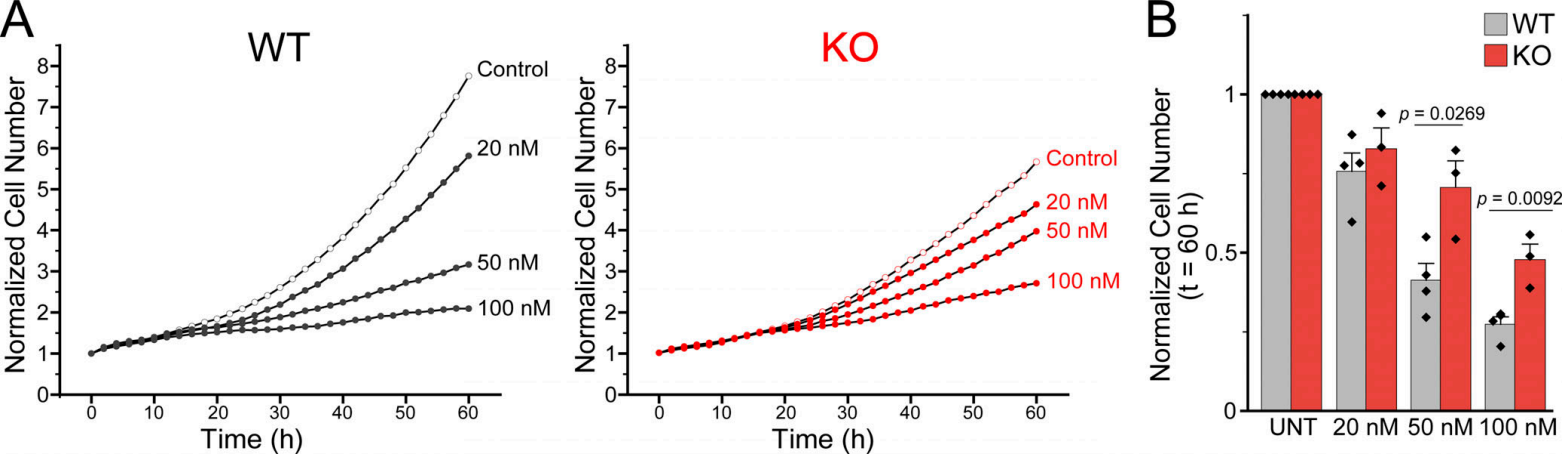
**Cerivastatin inhibits NSC proliferation in vitro in a dose dependent manner. (A)** Quantification of proliferation from live cell imaging of NSC proliferation as in Fig. 3 E, but with Cerivastatin added at timepoint 0. Data are from four images per each of three wells for each of *n* = 4 embryos for WT and *n* = 3 embryos for *Piezo1* KO. **(B)** Quantification of live-cell proliferation in the presence of cerivastatin after 60 h and normalized to untreated NSCs reveals that cerivastatin reduces proliferation of NSCs in a dose-dependent manner. *Piezo1* KO NSCs are less sensitive to drug treatment. P values determined by a two-sample *t*-test.

**Figure S6.**
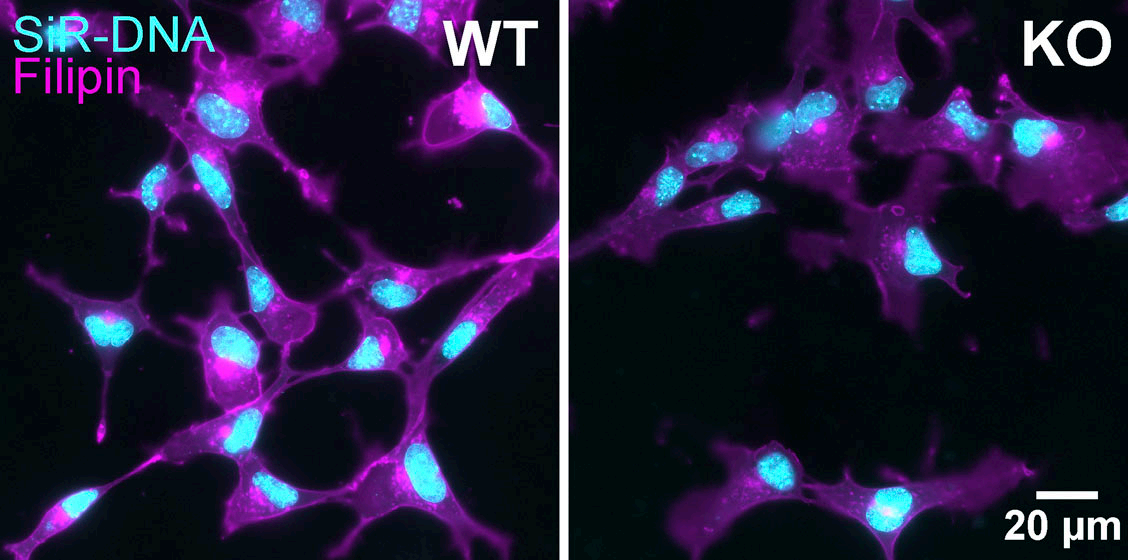
**Cholesterol is lower in *Piezo1* KO NSCs.** Images from Fig. 4 E of cultured E10.5 WT and *Piezo1* KO NSCs with Filipin III, shown here with nuclei counterstained with siR-DNA. *n* = 4 embryos for WT and *n* = 4 embryos for *Piezo1* KO from three independent experiments. Related to Fig. 4, E and F.

**Figure S7.**
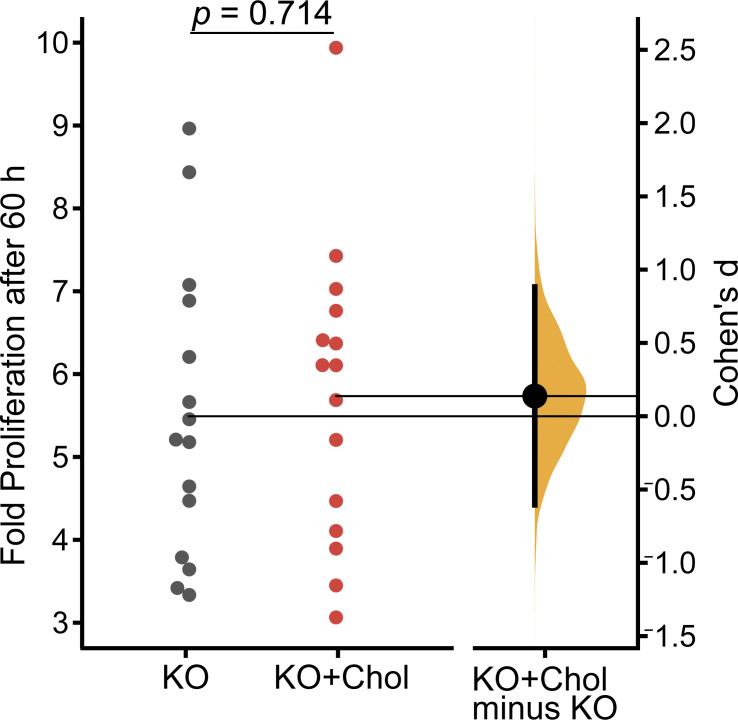
**Cholesterol supplementation does not rescue *Piezo1* KO NSC proliferation.** Quantitation of live-cell imaging of NSC proliferation. Gardner-Altman estimation plot of the fold proliferation after 60 h for *Piezo1* KO NSCs with and without cholesterol-MBCD treatment. Data are from four images per three wells for each of *n* = 15 samples from 7 embryos for WT and *n* = 15 samples from 7 embryos for *Piezo1* KO from eight independent experiments (Cohen’s *d* = 0.138).

**Figure S8.**
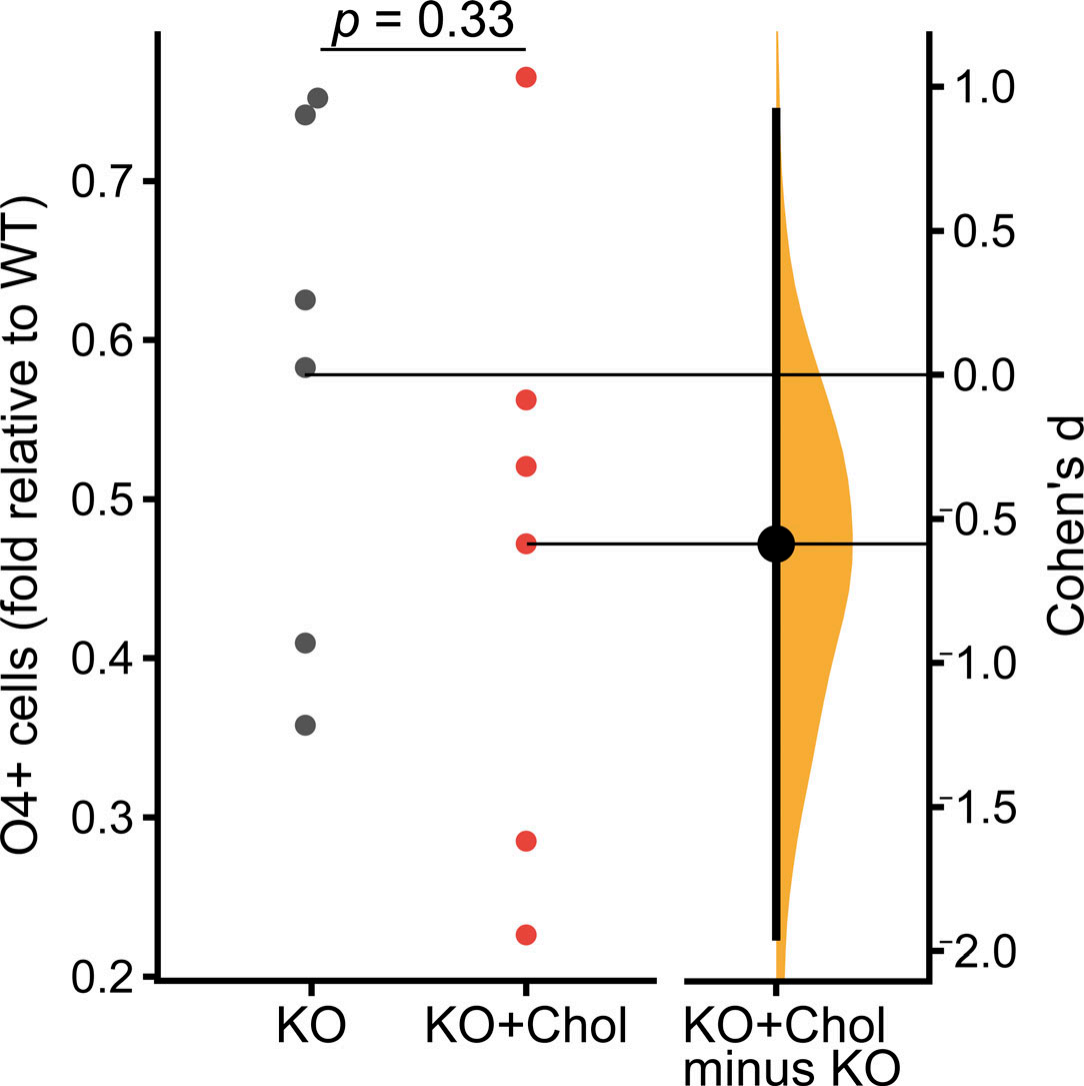
**Cholesterol supplementation does not rescue *Piezo1* KO NSC oligodendrocyte differentiation.** Gardner-Altman estimation plot of O4+ oligodendrocytes formed in *Piezo1* KO mNSCs with and without cholesterol-MBCD treatment. Data are normalized to untreated WT samples. *n* = 6 samples from 5 embryos, 25,704 cells quantified from 80 unique fields of view for WT, and *n* = 6 samples from 4 embryos, 30,301 cells quantified from 76 unique fields of view for *Piezo1* KO and 33,355 cells quantified from 60 unique fields of view for *Piezo1* KO + cholesterol from two independent experiments (Cohen’s *d* = −0.587). Related to Fig. 5.

Fig. S1 shows a lack of *Piezo1* mRNA expression in the *Piezo1* KO hindbrain. Fig. S2 shows disorganization of *Piezo1* KO hindbrain tissue with Nestin and Sox2 staining. Fig. S3 shows oligodendrocyte staining images of differentiated NSCs from WT and *Piezo1* KO. Fig. S4 shows the principle component analysis plot of brain RNAseq samples. Fig. S5 shows the effect of cerivastatin treatment on WT and *Piezo1* KO NSC proliferation. Fig. S6 shows filipin staining of WT and *Piezo1* KO with nuclei counterstained. Fig. S7 shows that cholesterol supplementation does not improve the proliferation of *Piezo1* KO NSCs. Fig. S8 shows that cholesterol supplementation does not improve oligodendrocyte differentiation of *Piezo1* KO NSC. Video 1 shows reduced neuronal differentiation in vivo of *Piezo1* KO E10.5 whole embryos with TUJ1 staining with lightsheet microscopy. Video 2 shows WT NSCs proliferate faster than *Piezo1* KO NSCs with live cell imaging. Table S1, tab 1 shows the results of differential gene expression analysis of RNAseq data from WT and *Piezo1* KO E10.5 brains. Table S1, tab 2 shows the expanded list of the canonical pathways identified by Ingenuity Pathway Analysis. Table S2 shows the canonical pathways affected in E10.5 *Piezo1* KO brains identified by Ingenuity Pathway Analysis (IPA). Table S3 lists the antibodies used in this study. Table S4 shows the microscope information used in this study.

## Results

### *Piezo1* KO alters brain morphology and neurogenesis

To establish the contributions of PIEZO1 in brain development, we used a constitutive *Piezo1* KO mouse model (Ranade et al., 2014). This mouse model demonstrated the importance of PIEZO1 in vascular development; however, brain architecture in the absence of *Piezo1* had not been examined. Due to the embryonic lethality of *Piezo1* KO mice, which begins after E9.5 and progresses through gestation (Ranade et al., 2014), we used E10.5 embryos—a time point that provides sufficient numbers of embryos to analyze while also allowing early stages of neural development to be examined. At this stage, the neural tube has closed and is composed primarily of NSCs and nascent neurons. The neuroepithelium displays pseudostratification and an apico-basal polarity of NSCs that is required for neurogenesis, neuronal migration, and the integrity of the adherens junctions at the inner/apical border (Kriegstein and Alvarez-Buylla, 2009; Paridaen et al., 2013; Veeraval et al., 2020).

**Figure 1.**
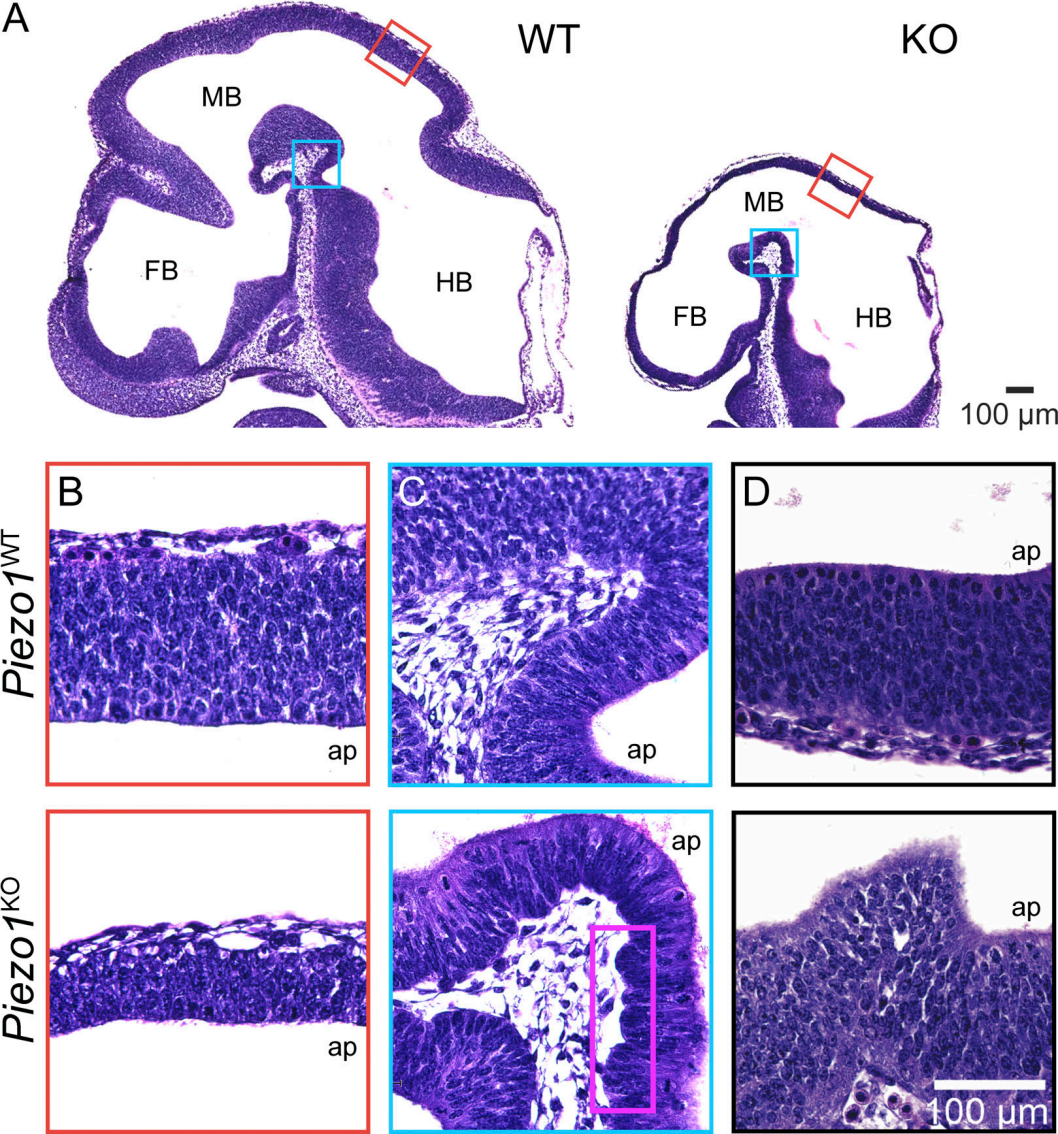
**Histological analysis reveals abnormalities in the developing brain of *Piezo1* KO mutant mice. (A)** Representative H&E stains of E10.5 WT (left) and *Piezo1* KO (right) littermate embryo sections. FB, forebrain; MB, midbrain; HB, hindbrain. Regions marked by red and blue boxes are shown at higher magnification in B and C. **(B–D)** Representative images highlighting differences in neuroepithelial thickness (B), inner/apical and outer/basal border morphology (C and D), and pseudostratified layering (D) between E10.5 WT (top row) and *Piezo1* KO (bottom row) littermates. Purple box in C highlights the abnormal undulations at the basal border in the mutant. Images in A–D are representative of *n* = 7 embryos for WT and for *Piezo1* KO. Scale bar in D also applies to B and C.

To determine the impact of *Piezo1* deletion on the morphology of the developing brain, we performed histological analysis through H&E staining of WT and *Piezo1* KO brains. As previously reported, we found that mutant embryos were considerably smaller than their WT counterparts (Ranade et al., 2014; Li et al., 2014; Fig. 1 A). In all regions of the developing brain (forebrain, midbrain, hindbrain), *Piezo1* KO embryos possessed a thinner neuroepithelium than WT littermates (Fig. 1, A and B). Furthermore, we observed unevenness at both the inner/apical and outer/basal borders in *Piezo1* KO brain sections, which were more pronounced in the midbrain and hindbrain (Fig. 1, C and D). We attributed the more pronounced phenotype in the mid- and hindbrain to the back-to-front temporal order of brain development, wherein the hindbrain, midbrain, and forebrain develop sequentially (Chen et al., 2017). In regions of notable border disruptions, the normal pseudostratification that is characteristic of the neuroepithelium was disrupted (Fig. 1 D). These findings suggest that *Piezo1* helps modulate the number and the organization of cells in the neuroepithelium and is involved in maintaining its integrity.

**Figure 2.**
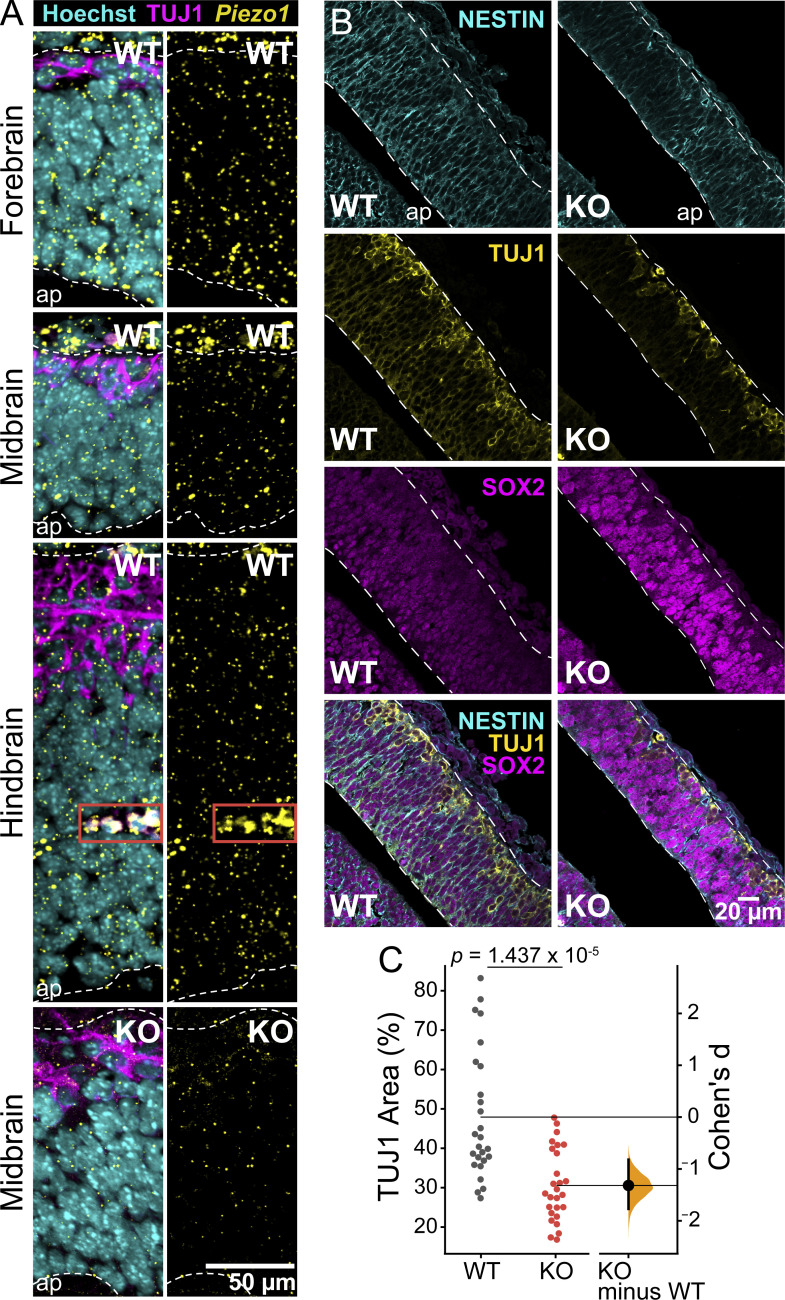
**Lack of *Piezo1* expression reduces differentiation in vivo. (A)** Fluorescent ISH of *Piezo1* mRNA in the E10.5 mouse brain. Representative ISH images of WT embryo sections for the forebrain, midbrain, and hindbrain regions shown in top three panels, and *Piezo1* KO mutant (midbrain) shown in the bottom panel. *Piezo1* mRNA signal shown in yellow, TUJ1 antibody staining of neurons shown in magenta, and Hoechst-stained nuclei in cyan. The white dashed lines demarcate the neuroepithelium, and the red solid box highlights a blood vessel; ap, apical/ventricular border. Images are representative of *n* = 3 embryos for WT and *n* = 2 embryos for *Piezo1* KO. **(B)** Representative WT and *Piezo1* KO midbrain regions from embryo sections immunostained with NESTIN (cyan), TUJ1 (yellow), and SOX2 (magenta). The white dashed lines demarcate the neuroepithelial borders. **(C)** Gardner-Altman estimation plots of the area of TUJ1+ regions normalized to the total area of the neuroepithelium for WT and *Piezo1* KO embryos from images as in B. Data are from *n* = 6 embryos (26 unique fields of view) for WT and *n* = 5 embryos (27 unique fields of view) for *Piezo1* KO from five independent experiments (P value calculated by two-sample *t*-test, Cohen’s *d* = −1.32). See also Video 1 and Fig. S2.

We next examined *Piezo1* mRNA expression patterns in the developing brain using ISH of brains isolated from WT E10.5 embryos. We observed a punctate expression of *Piezo1* spanning the width of the neuroepithelium in all brain regions—forebrain, midbrain, and hindbrain (Fig. 2 A). ISH of littermate *Piezo1* KO brains showed an absence of signal, confirming the specificity of the *Piezo1* signal in WT embryos (Fig. 2 A, bottom panel, and Fig. S1). As expected, high expression of *Piezo1* was also observed in nascent blood vessels in the hindbrain (Fig. 2 A, third panel), supporting previous reports of the importance of PIEZO1 in the developing vasculature (Ranade et al., 2014; Li et al., 2014).

To observe the organization and location of neurons in *Piezo1* KO and WT littermate E10.5 embryos, we turned to whole mount immunofluorescence of TUJ1, an early neuronal marker, followed by optical clearing and imaging with lightsheet microscopy (Video 1). In *Piezo1* KO embryos, we observed less TUJ1 staining in the brain as well as a reduction of axonal fibers (Video 1). Immunolabeling of tissue sections confirmed that *Piezo1* KO brains displayed a notably diminished TUJ1+ layer compared to WT (Fig. 2, B and C). In WT sections, immunostaining with the NSC markers NESTIN and SOX2 highlighted the alignment of NSCs along an apical to the basal axis, whereas in the *Piezo1* KO sections, this arrangement was disrupted (Fig. S2, insets). Notably, neurons localized correctly at the outer/basal region of the neuroepithelium, indicating that outward migration of early neurons along NSCs is not hindered in *Piezo1* KO developing brains. However, the diminished neuronal layer (Fig. 2, B and C; and Video 1) suggests that differentiation is compromised.

**Video 1.** **WT and *Piezo1* KO whole mount immunostaining of E10.5 embryos.** Embryos immunostained with TUJ1 (magenta), a marker for early neurons, and with Zona Occludens-1 (ZO-1; green), a protein enriched in adherens junctions at the borders of the neuroepithelium. Video frames represent lightsheet microscopy images collected at 5 µm slices. Related to Fig. 2 B.

### *Piezo1* KO reduces NSC proliferation and differentiation in vitro

**Figure 3.**
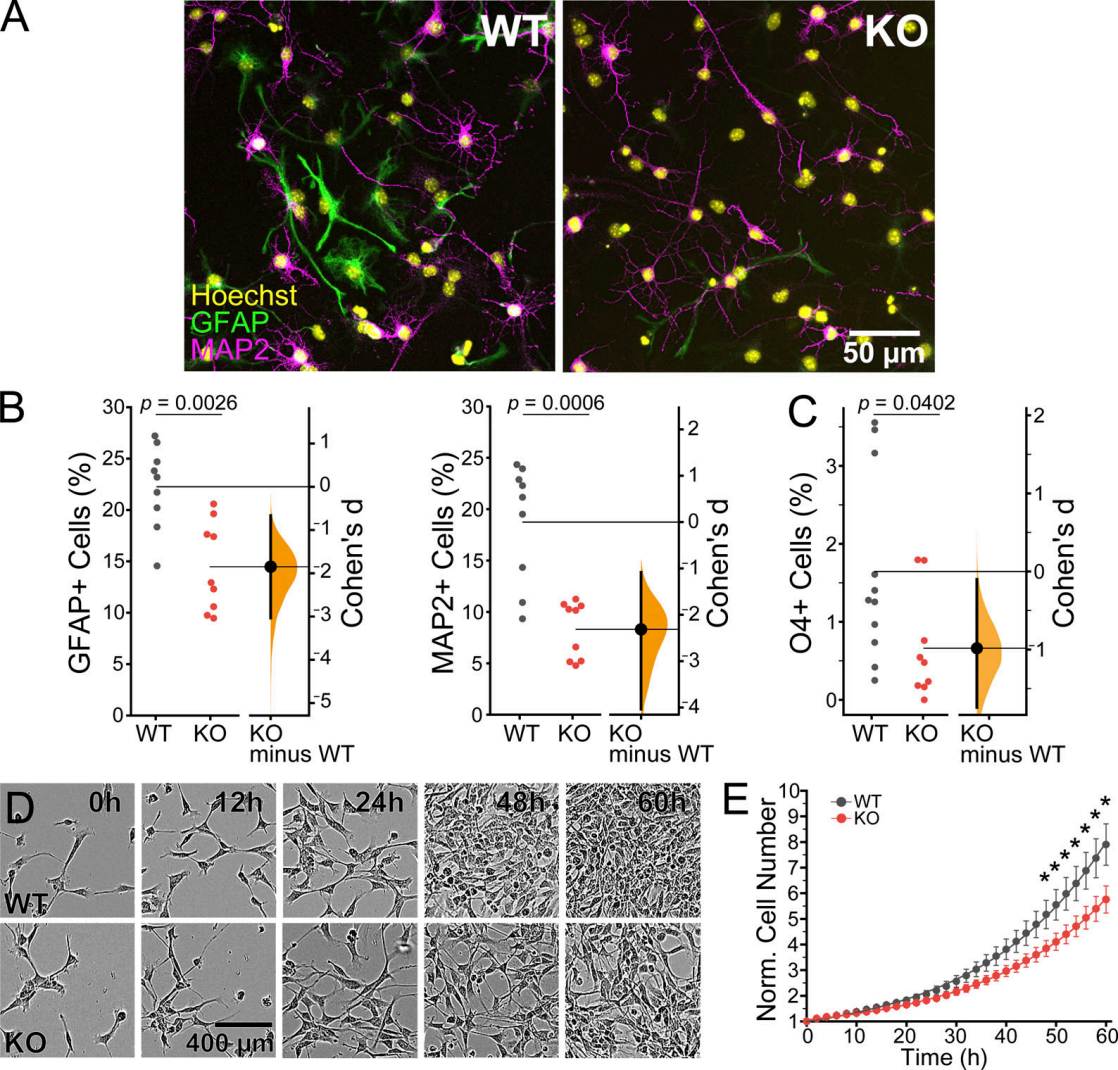
***Piezo1* KO reduces differentiation and proliferation in vitro. (A)** Representative fluorescence images of cultured E10.5 WT and *Piezo1* KO NSCs differentiated for 4 d in vitro and immunostained for astrocytic marker GFAP (green), neuronal marker MAP2 (magenta), and counterstained nuclei with Hoechst (yellow). **(B)** Gardner-Altman estimation plot of the percentage of GFAP+ (Cohen’s *d* = −1.85) and MAP2+ (Cohen’s *d* = −2.32) differentiated cells. *n* = 9 samples from 4 embryos for each of WT and for *Piezo1* KO from three independent experiments. 7,461 cells from 14 unique fields of view quantified for WT and 5,957 cells from 18 unique fields of view quantified for *Piezo1* KO. **(C)** Gardner-Altman estimation plots of the percentage of cells immunostained for the oligodendrocyte marker O4+ after 10 d of differentiation. See also Fig. S3. Data are from *n* = 11 samples from 6 embryos, 4,489 cells from 51 unique fields of view quantified for WT and *n* = 9 samples from 4 embryos, 3,941 cells from 54 unique fields of view quantified for *Piezo1* KO from two independent experiments (Cohen’s *d* = −0.984). **(D)** Representative live-cell phase-contrast images from proliferating WT and *Piezo1* KO NSCs imaged over 60 h. See also Video 2. **(E)** Quantitation of proliferation from live-cell imaging of NSC proliferation as shown in Video 2. Data are from four images per three wells for each of *n* = 4 embryos for WT and *n* = 4 embryos for *Piezo1* KO, P < 0.05 for all points after 48 h (from left to right: P value = 0.04847 at 48 h, 0.047 at 50 h, 0.04246 at 52 h, 0.04451 at 54 h, 0.04067 at 56 h, 0.03997 at 58 h, 0.03733 at 60 h as determined by two-sample *t*-test).

A regulated balance of neural stem cell proliferation and differentiation occurs as the brain develops. The neuroepithelial layer expands and thickens as the NSCs proliferate while a subset of daughter cells differentiate into neurons and migrate outward to the outer/basal region. To examine the role of PIEZO1 on neural stem cell fate more closely, we turned to in vitro cultures of NSCs. We isolated and expanded NSCs from WT and *Piezo1* KO E10.5 brains and differentiated them by withdrawing growth factors for 4 d to examine neuron and astrocyte formation or for 10 d to examine oligodendrocyte formation. The differentiated cells were immunostained for relevant markers—GFAP as a marker for astrocytes, MAP2 as a marker for neurons (Fig. 3 A), and O4 as a marker for oligodendrocytes (Fig. S3). The percentage of differentiated cells was quantified and revealed a reduction of neurons and astrocytes (Fig. 3 B) and oligodendrocytes (Fig. 3 C and Fig. S3) in *Piezo1* KO cells. Taken together, these results demonstrate that the lack of PIEZO1 in mouse NSCs compromises differentiation into all three neural lineages.

To determine whether the thinner neuroepithelium observed in *Piezo1* KO E10.5 embryos is due to a role of PIEZO1 in NSC proliferation, we examined the proliferation of cultured WT and *Piezo1* KO NSCs in real-time using live-cell microscopy (Fig. 3, D and E; and Video 2). We found that *Piezo1* KO mNSCs have reduced proliferation rates; after 60 h in culture, WT cells had proliferated 7.9-fold while *Piezo1* KO cells had proliferated 5.8-fold, yielding a doubling time of 20 h, 7 min for WT and 23 h, 45 min for *Piezo1* KO i.e., an 18% lower doubling time of *Piezo1* KO mNSCs (Fig. 3 E). This reduction in proliferative capacity may lead to fewer NSCs comprising the E10.5 neuroepithelium, thus leading to the observed thinner neuroepithelium in vivo (Fig. 1 B).

**Video 2.** **Piezo1 KO reduces proliferation of E10.5 NSCs in vitro.** Time-lapse fluorescence and phase contrast imaging of WT and *Piezo1* KO NSCs over 60 h. Top: Nuclei stained with siR-DNA (red). Bottom: Phase contrast imaging overlaid with nuclear stain. Images collected every 2 h. Video frame rate is 3 frames/s. Representative images from four images per three wells for each of *n* = 4 embryos for WT and *n* = 3 embryos for *Piezo1* KO. Related to Fig. 3, D and E.

### *Piezo1* KO reduces the expression of cholesterol synthesis genes

**Figure 4.**
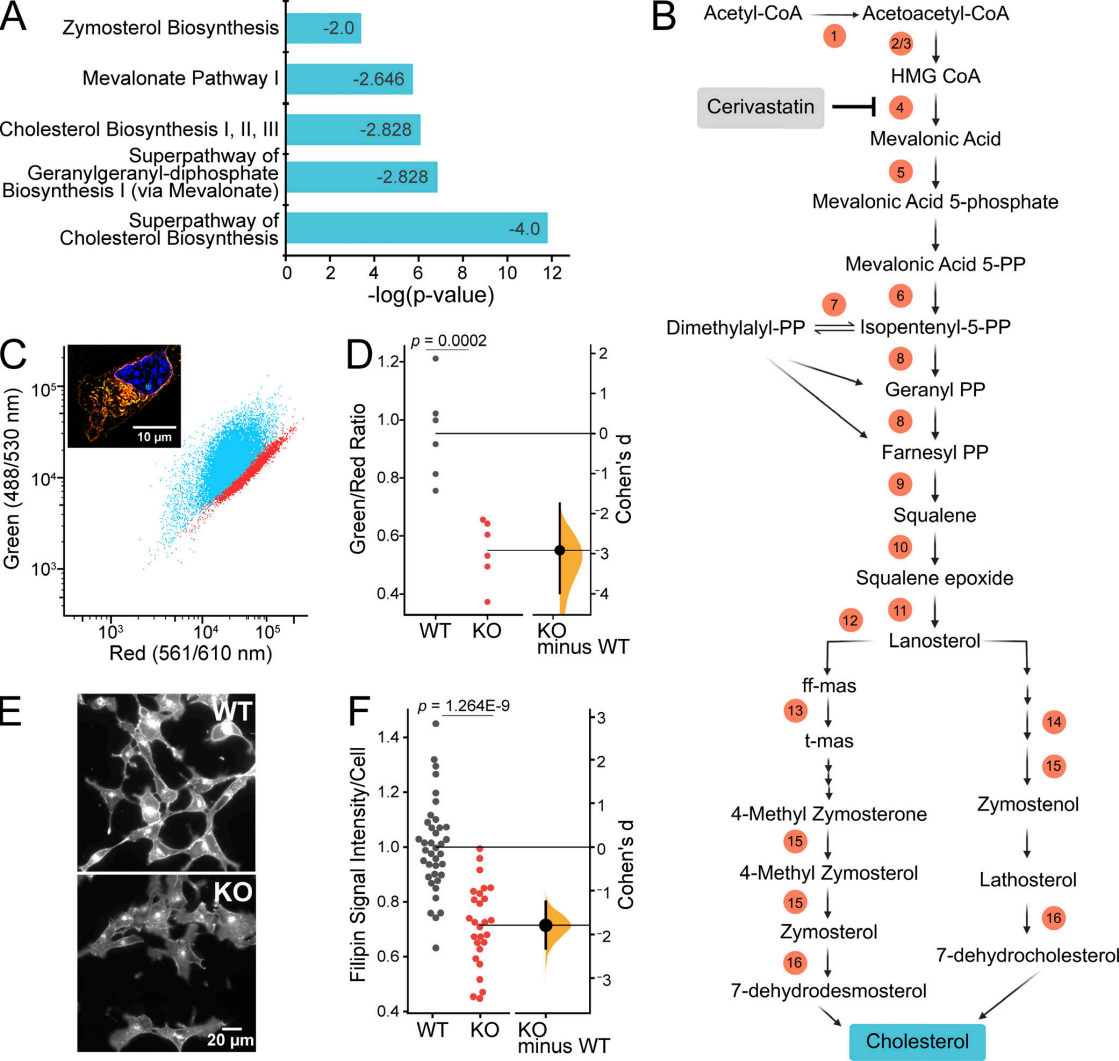
***Piezo1* KO results in downregulation of cholesterol biosynthesis. (A)** Downregulation of cholesterol biosynthesis, the most predominant effect of *Piezo1* KO identified by Ingenuity Pathway Analysis of differential gene expression data of WT and *Piezo1* KO E10.5 brains. See also Table S2. *Z*-score is indicated inside the bar for each pathway. **(B)** Schematic of the cholesterol biosynthesis pathway. Circled numbers indicate the 16 enzyme genes within the pathway downregulated by 1.5-fold or more in *Piezo1* KO brains relative to WT. ff-mas, 14-demethyl-14-dehydrolanosterol; t-mas, 14-demethyl-lanosterol. Numbers correspond to the following list: (enzyme, gene name, Log2Ratio) *1,* acetyl-CoA acetyltransferase 2, *Acat2*, −1.00; *2,* HMG-CoA synthase 1, *Hmgcs1*, −1.24; *3,* HMG-CoA synthase 2, *Hmgcs2*, −1.67; *4,* HMG-CoA reductase, *Hmgcr*, −0.74; *5,* Mevalonate kinase, *Mvk*, −0.72; *6,* Mevalonate-5-pyrophosphate decarboxylase, *Mvd*, −0.95; *7,* Isopentenyl-5-isomerase, *Idi1*, −1.45; *8,* Farnesyl diphosphate synthase, *Fdps*, −1.64; *9,* Squalene synthase, *Fdft1*, −0.98; *10,* Squalene epoxidase, *Sqle*, −0.61; *11,* Lanosterol synthase, *Lss,* −0.82; *12,* Cytochrome P450 subfamily member A1, *Cyp51* −0.84; *13,* Transmembrane 7 superfamily, *Tm7sf2*, −0.98; *14,* Methosterol monooxygenase, *Msmo*, −1.25; *15,* Hydroxysteroid 17 beta-dehydrogenase, *Hsd17b7*, −0.81; *16,* Sterol C-5 desaturase, *Sc5d*, −0.78. Cerivastatin (gray box) is an inhibitor of HMG-CoA reductase, the rate-limiting enzyme of the cholesterol biosynthesis pathway. **(C)** Representative scatter plot of flow cytometry analysis of Nile Red stained WT and *Piezo1* KO NSCs. WT (blue), *Piezo1* KO (red). Inset: Representative overlay image of both green and red channels of Nile Red stained images of WT NSCs grown in proliferative conditions. Nuclei are counterstained with Hoechst (blue). **(D)** Gardner-Altman estimation plot of data as in C of Nile Red staining of NSCs plotting the ratio of the geometric mean fluorescence obtained from the green and red channels of WT and *Piezo1* KO NSCs (Cohen’s *d* = −2.92). Data are from *n* = 6 samples from 3 embryos for WT and for *Piezo1* KO and are representative of two experimental replicates. **(E)** Representative images of WT and *Piezo1* KO NSCs grown in proliferative conditions and stained with Filipin III (see also Fig. S6). **(F)** Gardner-Altman estimation plot of data as in E of Filipin III staining of NSCs plotting the fluorescence intensity per cell in images of WT and *Piezo1* KO NSCs (Cohen’s *d* = −1.79). Data from *n* = 4 embryos, 899 cells from 38 unique fields of view for WT and *n* = 4 embryos, 729 cells from 27 unique fields of view quantified for *Piezo1* KO from three experimental replicates.

For an unbiased approach to illuminate the mechanisms leading to the observed *Piezo1* KO phenotypes, we turned to gene expression analysis. We isolated RNA from E10.5 WT and *Piezo1* KO brains for bulk RNAseq and performed differential gene expression analysis, identifying 8,609 genes that are differentially regulated (Table S1). PCA, which provides a quality assessment of the data, shows that samples cluster by genotype but show some variance based on sex (Fig. S4). Moreover, the *Piezo1* KO samples are less tightly clustered than WT samples, which we attribute to variability in the progression of lethality (ranging from E9.5 to birth; Li et al., 2014; Ranade et al., 2014) and difference in the severity of phenotype across embryos. When we examined canonical pathways affected by *Piezo1* KO, considering only genes altered by >1.5-fold in expression, integrin signaling and actin cytoskeleton emerged in the list of differentially regulated pathways (Table S1, tab 2), consistent with previously reported interplay between integrins, cytoskeleton, and Piezo1 (McHugh et al., 2010; McHugh et al., 2012; Nourse and Pathak, 2017). However, the most striking results were a widespread downregulation in *Piezo1* KO brains of genes in the pathways related to cholesterol biosynthesis, which includes genes responsible for the synthesis of mevalonate, geranylgeranyl diphosphate, and zymosterol (Fig. 4 A and Table S2). Sixteen genes along the cholesterol biosynthesis pathway were affected (Fig. 4 B and Table S2). Furthermore, two additional genes in the pathway were also downregulated to a lesser extent, *Dhcr7*, encoding 7-dehydrocholesterol reductase (−1.46-fold) and *Pmvk*, encoding phosphomevalonate kinase (−1.34-fold; Table S2). In addition, several genes encoding enzymes that catalyze the oxidation and esterification of cholesterol are upregulated: *Cyp46a1* (1.78-fold), *Ch25h* (2.09-fold), *Cyp27a1* (1.44-fold), and *Soat2* (19.2-fold), which would further contribute to the reduction of free cholesterol.

The gene *Hmgcr*, encoding HMG-CoA reductase, the rate-limiting enzyme for the cholesterol biosynthetic pathway, was downregulated by 1.7-fold in the brains of *Piezo1* KO mice (Fig. 4 B and Table S1). Statin drugs inhibit HMGCR activity by binding to the catalytic site of the enzyme (Istvan, 2003). We treated WT NSCs with cerivastatin to mimic a reduction in cholesterol biosynthesis and then examined cell numbers using live-cell imaging. We found that cerivastatin reduced proliferation in a dose-dependent manner, and when WT cells were treated with 20 nM cerivastatin, cell proliferation rates equaled that of *Piezo1* KO cells (Fig. S5 A). Higher doses of cerivastatin elicited greater reduction of proliferation, with 100 nM reducing WT NSC proliferation by ∼75% (Fig. S5 B). Cerivastatin treatment also inhibited *Piezo1* KO cell proliferation but to a lesser extent (Fig. S5), likely since HMGCR levels are already diminished. These observations suggest that cholesterol biosynthesis contributes to sustaining the proliferation of NSCs.

Cholesterol is an essential component of cell membranes, specifically of lipid raft microdomains that are important cell signaling hubs for cell proliferation, neuronal differentiation, synaptogenesis, and oligodendrocyte maturation (Hussain et al., 2019; Saher et al., 2005). The de novo cholesterol synthesis pathway is also critical for brain development since cholesterol is the precursor for steroid hormones, which are important for the structural organization of the brain (Kawata, 1995). The downregulation of multiple genes related to cholesterol synthesis and upregulation of genes related to cholesterol oxidation and esterification suggests that PIEZO1 contributes to cholesterol metabolism in NSCs.

### *Piezo1* KO lowers neutral lipids and cholesterol levels in NSCs

Cholesterol is a neutral lipid which comprises 20–40% of cell membranes and is stored in intracellular lipid droplets (Meyers et al., 2017; Farmer et al., 2020). Therefore, we explored whether reduced expression of cholesterol biosynthetic genes in *Piezo1* KO NSCs translates into changes in cellular lipid composition. Nile Red is a fluorescent probe that shows large emission shifts when in different hydrophobic environments (Greenspan et al., 1985; Brown et al., 1992). A metric for characterizing Nile Red signal in cells is the ratio of its green fluorescence, which reflects Nile Red binding to neutral lipids (e.g., cholesterol and triacylglycerols), to its red fluorescence, which reflects binding to phospholipids (Klymchenko and Kreder, 2014; Diaz et al., 2008). We first verified appropriate staining of cellular lipids by Nile Red in NSCs by visualizing its fluorescence with microscopy. As expected, we observed both red and green fluorescence upon Nile Red labeling of cells (Fig. 4 C, inset). To quantitatively compare Nile Red signal between WT and *Piezo1* KO, we next analyzed green and red fluorescence emanating from Nile Red–labeled NSCs by flow cytometry. Similar to the microscopy images, we observed both green and red fluorescence signals in these cells, with WT showing a higher intensity of green fluorescence compared to *Piezo1* KO, and correspondingly, a larger green-to-red fluorescence ratio (Fig. 4, C and D), thereby indicating that *Piezo1* KO reduces membrane neutral lipid.

Next, we examined cholesterol levels in NSCs by staining with Filipin III, a cholesterol-binding fluorescent compound that allows visualization and quantitation of free cholesterol (i.e., unesterified cholesterol) in cellular membranes (Maxfield and Wüstner, 2012). Analysis of Filipin III fluorescence in NSCs indicated that *Piezo1* KO cells have ∼26% less signal than WT cells (Fig. 4, E and F; and Fig. S6). This demonstrates that *Piezo1* KO results in a reduction of free cholesterol in NSCs.

### Cholesterol supplementation improves *Piezo1* KO NSC differentiation

We then asked whether the addition of cholesterol could rescue the in vitro phenotypes of reduced proliferation and differentiation in *Piezo1* KO NSCs. Live-cell proliferation assays performed in the presence of 10 µg/ml water-soluble cholesterol-methyl-β-cyclodextrin (cholesterol–MBCD, see Materials and methods), did not significantly improve *Piezo1* KO proliferation (Fig. S7).

**Figure 5.**
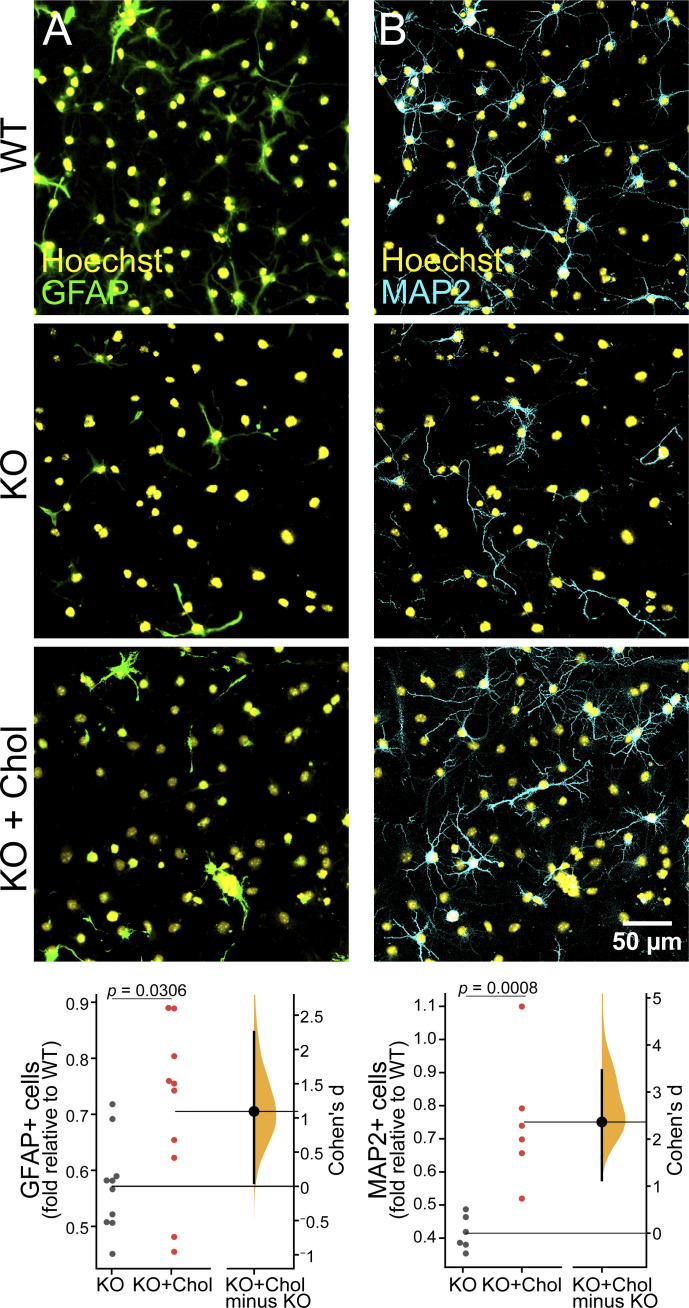
***Piezo1* KO NSC differentiation is partially rescued by exogenous cholesterol. (A)** Representative fluorescence images of WT and *Piezo1* KO NSCs differentiated for 4 d and immunostained for GFAP (green) and nuclei stained with Hoechst (yellow). Cells were either maintained in standard differentiation media (top panels) or media supplemented with 10 µg/ml cholesterol-MBCD (bottom panel). Bottom row shows a Gardner-Altman estimation plot of images as in A shows increased astrocyte differentiation with cholesterol-MBCD supplementation of *Piezo1* KO NSCs. Data are normalized to untreated WT samples. *n* = 7 embryos, 9,797 cells from 24 unique fields of view quantified for WT, and *n* = 7 embryos, 12,020 cells from 39 unique fields of view quantified for *Piezo1* KO and 11,761 cells from 37 unique fields of view quantified for *Piezo1* KO + cholesterol from four independent experiments (Cohen’s *d* = 1.09). **(B)** Representative fluorescence images of WT and *Piezo1* KO NSCs differentiated for 4 d and immunostained for MAP2 (cyan) and nuclei-stained with Hoechst (yellow). Cells were either maintained in standard differentiation media (top panels) or media supplemented with 10 µg/ml cholesterol-MBCD (bottom panel). Gardner-Altman estimation plot of images as in B shows increased neuronal differentiation with cholesterol supplementation of *Piezo1* KO NSCs. Data are normalized to untreated WT samples. For GFAP, *n* = 14 samples from 7 embryos, 9,797 cells from 24 fields of view; for WT, *n* = 10 samples from 7 embryos, 12,020 cells from 39 fields of view; for *Piezo1* KO, *n* = 10 from 7 embryos, 11,761 cells from 37 fields of view for *Piezo1* KO + cholesterol from four independent experiments (Cohen’s *d* = 1.09). For Map2, *n* = 6 samples from 4 embryos, 7,461 cells from 14 unique fields of view quantified for WT and *n* = 6 samples from 4 embryos, 5,987 cells from 18 unique fields of view quantified for *Piezo1* KO and 5,414 cells from 16 unique fields of view for *Piezo1* KO + cholesterol from two independent experiments (Cohen’s *d* = 2.36).

We then performed differentiation assays in media supplemented with 10 µg/ml cholesterol–MBCD and examined the formation of neurons, astrocytes, and oligodendrocytes (Fig. 5). While oligodendrocyte differentiation was not affected (Fig. S8), cholesterol-MBCD addition increased astrocytic differentiation by 1.25-fold (Fig. 5 A) and neuronal differentiation by 1.8-fold (Fig. 5 B). Thus, cholesterol supplementation partially rescued the phenotype of *Piezo1* KO NSCs in vitro.

Collectively, our results indicate that the PIEZO1 channel influences cellular cholesterol metabolism necessary for neural development.

## Discussion

### *Piezo1* knockout results in aberrant neuroepithelial development

Roles for PIEZO1 in the neural lineage include directing lineage choice of human fetal NSCs in vitro (Pathak et al., 2014), axonal guidance (Koser et al., 2016) and regeneration (Song et al., 2019), and progenitor cell aging of adult rat oligodendrocyte progenitors (Segel et al., 2019). However, PIEZO1’s role in mammalian embryonic brain development has not yet been addressed*.* Here, we examined its contribution to brain development using constitutive *Piezo1* KO mice. We report *Piezo1* expression throughout the fore-, mid-, and hindbrain, elucidating its expression pattern in the embryonic neuroepithelium (Fig. 2 A)*.* We show that PIEZO1 influences the structure and integrity of the neuroepithelium (Figs. 1, B–D; 2 B; and S1), and identified a new molecular pathway contributing to these phenotypes.

Previous work with constitutive knockout of *Piezo1* demonstrated a role for PIEZO1 in vascular development (Ranade et al., 2014; Li et al., 2014), raising the question of whether our observed phenotypes may be secondary to vascular disruption. Vascularization of the neuroepithelium initiates from the outer/basal surface around E9.5–E10.5 and continues until birth (Paredes et al., 2018). Between E8.5 and E10.5, a blood vessel network forms surrounding the neural tube, and subsequently, new vessels sprout from this network and begin to invade the neuroepithelium; by birth, the neural tissue is extensively vascularized. The phenotypes we observe in the neuroepithelium at E10.5 thus precede significant brain vascularization. Additionally, recapitulation of the key phenotypes—reduced proliferation and differentiation of NSCs—in cultured NSCs in vitro demonstrate an effect of *Piezo1* KO on NSC biology that is independent of the channel’s role in vasculature development. Emerging studies reveal a reciprocal relationship between the development of the vasculature and of neural cells (Paredes et al., 2018); thus, it is possible that as the invasion of blood vessels progresses through gestation, the vasculature tissue influences the neural compartment, which may give rise to additional neurodevelopmental phenotypes not captured by our study.

We present several lines of evidence that PIEZO1 is important for NSC proliferation and differentiation. First, we find that the neuroepithelium of *Piezo1* KO brains is significantly thinner than that of WT littermate embryos, implying that the proliferation of NSCs is compromised in these animals (Figs. 1 B and 2 B). In addition, the doubling time of E10.5 NSCs in vitro decreased by 18% (Fig. 3, D and E; and Video 2). Over the course of embryonic brain development, this reduced proliferation rate would result in a significantly lower number of cells and therefore a thinner neuroepithelium, consistent with our observations from H&E staining of E10.5 embryo sections. We also show that differentiation of the NSCs into neurons is impaired in *Piezo1* KO brain sections (Fig. 2 B and Video 1). Second, we show that *Piezo1* KO NSC differentiation is reduced in vitro*,* indicating that these effects are cell autonomous to NSCs. Differentiation of all cell types from NSCs is decreased in the absence of *Piezo1* (Fig. 3, A–C). In our previous study with human fetal NSCs, *Piezo1* siRNA-mediated knockdown resulted in a decreased neuronal but increased astrocytic specification (Pathak et al., 2014). Both studies find that reduced PIEZO1 function decreases neuron production. The differences in astrocyte differentiation between our previous study and the current one may be a reflection of biological (human versus rodent) and/or technical differences. Our previous study used siRNA-mediated knockdown which causes an incomplete and transient reduction in expression, while our present study uses a constitutive genetic knockout. It is also possible that the differences arise from differing levels of PIEZO1 activity. Future studies employing constitutive genetic *PIEZO1* KO in human cells may help address these differences.

### Nexus between cellular mechanics and cholesterol metabolism

We find a remarkable downregulation of 16 genes by at least 1.5-fold in the cholesterol biosynthesis pathway in *Piezo1* KO brains (Fig. 4 B and Table S1). Additionally, four other genes that metabolize cholesterol are upregulated. Our in vitro experiments (Figs. 3, 4, and 5) support the model that the phenotypes observed are due to NSC-intrinsic defects in cholesterol biosynthetic pathway caused by *Piezo1* KO. Nile Red staining indicates a reduction of neutral lipids (Fig. 4, C and D), and Filipin III staining of *Piezo1* KO NSCs indicates a reduction of unesterified (free) membrane cholesterol (Fig. 4, E and F; and Fig. S6). Furthermore, *Piezo1* KO NSCs supplemented with cholesterol in vitro show improved neuronal and astrocytic differentiation demonstrating the importance of reduced cholesterol in contributing to the observed phenotypes (Fig. 5).

Cholesterol is integral to cell membrane composition and the formation of lipid rafts, which serve as critical functional signaling hubs for many cell surface receptors. It functions as a covalent ligand for the hedgehog morphogenic proteins required for brain development and serves as a precursor for steroid hormones (Lewis et al., 2001; Hussain et al., 2019). Cholesterol directly binds to many membrane proteins (Hulce et al., 2013), including PIEZO1. Interestingly, some cholesterol binding sites have been mapped to disease-causing mutations in PIEZO1 (Buyan et al., 2020), and cholesterol modulates the electrical activity of PIEZO1 (Ridone et al., 2020).

Cholesterol plays a critical role in mammalian embryonic brain development as evidenced by human brain defects caused by inborn errors in cholesterol biosynthesis genes and phenotypes observed in mouse models (Hussain et al., 2019; Driver et al., 2016; Saito et al., 2009; Tozawa et al., 1999). For instance, mutations in *SC5D* encoding Sterol C-5 Desaturase (enzyme 16 in Fig. 4 B; downregulated 1.75-fold in *Piezo1* KO brains) cause lathosterolosis, a condition resulting in multiple congenital anomalies and intellectual disability (Brunetti-Pierri et al., 2002). Mutations in *DHCR7*, encoding the penultimate enzyme in the pathway (downregulated 1.46-fold in *Piezo1* KO brains) underlie Smith–Lemli–Opitz syndrome (SLOS), which includes symptoms of microcephaly, mental developmental delay, and hearing impairment (Irons et al., 1993). Furthermore, a mutation in the *Hsd17b7* gene (enzyme 15 in Fig. 4 B; downregulated 1.75-fold) causes precocious neural differentiation, abnormal interkinetic nuclear migration, and abnormal migration during NSC proliferation in mice (Driver et al., 2016).

Conditional knockout in NSCs of *Fdft1,* which encodes squalene synthase (enzyme 9 in Fig. 4 B; downregulated 1.88-fold), results in smaller brains, thinner neuroepithelium, and reduced neurons (Saito et al., 2009). We observed a similar phenotype to that of the *Fdft1* KO model, namely: smaller brains, a thinner neuroepithelium, and a reduced neuronal layer in *Piezo1* KO embryos. In *Fdft1* knockout mouse brains, reduction of cholesterol causes an increase in VEGF levels which increases blood vessel formations at E13.5 as a compensatory mechanism for raising cholesterol levels in the developing brain through lipoprotein uptake from blood (Saito et al., 2009). Interestingly, we also observe a strong upregulation of *Vegf* (3.5-fold, Table S1) in E10.5 *Piezo1* KO brains. Due to the embryonic lethality of the *Piezo1* KO mice, we were unable to examine whether *Piezo1* KO brains show a compensatory increase in the vascular invasion at subsequent time points. Nonetheless, the similarities in the phenotypes of the *Fdft1* and *Piezo1* knockouts suggest mechanistic commonalities related to cholesterol in early brain development and substantiate the idea that cholesterol downregulation downstream of *Piezo1* KO contributes to the observed neurodevelopmental defects.

### Mechanistic alternatives and study limitations

Our study strongly supports the hypothesis that *Piezo1* regulates neural development through de novo cholesterol biosynthesis but does not preclude other metabolites produced in the cholesterol synthesis pathway from also contributing to *Piezo1* KO phenotypes. Since transcripts encoding many enzymes in the cholesterol biosynthesis pathway are downregulated by *Piezo1* KO, this would lead to a reduction in several intermediate metabolites along the pathway, e.g., mevalonate, sterols, and the isoprenoids, geranylgeranyl diphosphate and farnesyl pyrophosphate. Some of these metabolites serve as precursors for steroid hormones, heme A, ubiquinone, and dolichol, which have important roles during brain development. For example, the steroid hormone vitamin D3 is important for NSC proliferation and differentiation into oligodendrocytes (Shirazi et al. 2015).

Our observation that WT NSC proliferation is more sensitive than *Piezo1* KO NSCs to cerivastatin, which targets HMG-CoA reductase (enzyme 4 in Fig. 4 B; downregulated 1.7-fold), suggests that cholesterol and/or its intermediate metabolites support proliferation (Fig. S5). Interestingly, we did not find cholesterol supplementation to rescue the decreased proliferation of *Piezo1* KO NSCs (Fig. S7), further supporting the idea that the intermediate metabolites may contribute to NSC proliferation. Indeed, among other cell types, mevalonate has been shown to promote Yap/Taz activation, which is the key regulator of cell proliferation (Sorrentino et al., 2014).

Our study does not exclude a role for *Piezo1* knockout impacting other relevant pathways. For instance, our RNAseq dataset shows upregulation of integrins, previously observed to be regulated by PIEZO1 (McHugh et al., 2012; McHugh et al., 2010; Nourse and Pathak 2017; Albarrán-Juárez et al., 2018; Atcha et al., 2021b). In addition, extracellular matrix components such as laminins, collagens, and fibronectin are also upregulated (Table S1), which may alter tissue mechanics and the cues that contribute to brain development. While integrin signaling and actin cytoskeleton emerged in the list of differentially regulated pathways (Table S1, tab 2) as would be expected based on previous findings in other systems, the effects were not as high as for the cholesterol-related pathways—e.g., only 11% of genes in the actin cytoskeletal signaling and 10% of genes in integrin signaling pathways were affected (see Table S1, tab 2, Ratio); the overall z-scores were also lower and the P values higher. In contrast, 44–67% of genes in 7 pathways associated with cholesterol biosynthesis are downregulated with high z-scores and low P values.

The absence of *Piezo1* likely leads to compensatory mechanisms, as suggested by the upregulation in our RNAseq dataset of *Vegf*, nutrient transporters (i.e., solute carrier family members, such as *Slc7a1*, *a3*, *a11*, *Slc6a9*), cholesterol transporters (*ApoE*, *Vldlr*), and *Piezo2* (Table S1, tab 1). Nevertheless, the link to cholesterol metabolism is novel and intriguing, and the molecular pathways and biophysical mechanisms linking *Piezo1* function to lipid metabolism are not mechanistically obvious, opening up a new line of investigation on how *Piezo1* expression and activity modulates cholesterol biosynthesis.

### A potential feedback mechanism between cholesterol and PIEZO1

PIEZO1 channel activation by membrane tension (Lewis and Grandl 2015; Syeda et al., 2016) is consistent with the force-from-lipids model of channel activation, and PIEZO1 activation sensitivity and inactivation are modulated by the local concentration in the membrane of fatty acids (e.g., margaric acid, arachidonic acid, and docosahexaenoic acid) as well as cholesterol (Ridone et al., 2020; Romero et al., 2019; Richardson et al., 2022). The addition of saturated fatty acids (e.g., margaric acid) increases membrane stiffness and decreases PIEZO1 activation, while the addition of polyunsaturated fatty acids (e.g., docosahexaenoic acid and eicosapentaenoic acid) lowers membrane stiffness and modulates channel inactivation in more complex ways (Romero et al., 2019). The cholesterol content of the membrane modulates both the channel activation sensitivity as well as inactivation kinetics (Ridone et al., 2020). Coarse-grain molecular dynamics simulations suggest that cholesterol and margaric acid interact with PIEZO1 through many of the residues associated with PIEZO1 channelopathies (Buyan et al., 2020). Together, these studies illuminate the importance of membrane composition and cholesterol to functional outcomes of PIEZO1 activity.

Our finding that *Piezo1* KO reduces cholesterol synthesis and alters lipid composition suggests the existence of a bidirectional feedback mechanism: PIEZO1 function enhances cholesterol biosynthesis whose presence in the membrane can, in turn, modulate channel activity. More broadly, our finding that *Piezo1* KO results in the downregulation of cholesterol biosynthesis uncovers a novel link between cellular mechanotransduction and cholesterol metabolism. This finding is not only relevant to brain development but also opens up new lines of investigation for examining a potential contribution of PIEZO1 in neurodegenerative diseases impacted by cholesterol dysregulation (Dai et al., 2021), such as Alzheimer’s disease, Parkinson’s disease, and Huntington’s disease.

## Supplementary Material

Table S1 contains 1 Differentially expressed genes in Piezo1 KO E10.5 brains compared to WT and an expanded list of canonical pathways with *z*-score of ±2 and IPA P value < 0.05

Table S2 lists changes in canonical pathways in E10.5 *Piezo1* KO brains identified by IPA

Table S3 provides a list of antibodies used in this study

Table S4 lists the microscopes used.

## Data Availability

RNA-sequencing data have been deposited in Gene Expression Omnibus (GEO) database, accession number GSE193099.

## Data availability

RNA-sequencing data have been deposited in Gene Expression Omnibus (GEO) database, accession number GSE193099.
