# Supplementary material for: Piezo1 regulates cholesterol biosynthesis to influence neural stem cell fate during brain development
Source: J Gen Physiol. 2022 Sep 7;154(10):e202213084. doi: 10.1085/jgp.202213084 (PMC9458470; doi:10.1085/jgp.202213084)
Supplement: Table S2 — lists changes in canonical pathways in E10.5 Piezo1 KO brains identified by IPA [file JGP_202213084_TableS2.docx]

**Table S2.** **Changes in Canonical Pathways in E10.5 *Piezo1* KO brains identified by Ingenuity Pathway Analysis (IPA).** Table shows pathways with z-score at +/- 2, and an IPA pathway *p*-value < 0.00015 (-log (*p*-value))**.** Only genes with log2 ratio of +/- 0.6 and pAdj<0.0001 are considered for the IPA analysis. Cholesterol biosynthesis-related pathways are marked in bold. The significance of the association between the data set and the canonical pathway was determined based on two parameters: (1) a *p-*value calculated using Fisher's exact test determining the probability that the association between the genes in the data set and the canonical pathway is due to chance alone and (2) a ratio of the number of genes from the data set that map to the pathway divided by the total number of genes that map to the canonical pathway.

| **Ingenuity Canonical Pathways** | **-log(*p*-value)** | **Ratio** | **z-score** | **Molecules/genes** |
| --- | --- | --- | --- | --- |
| **Superpathway of Cholesterol Biosynthesis** | **12.5** | **0.552** | **-4** | **ACAT2,CYP51A1,FDFT1,FDPS,HMGCR,HMGCS1,HMGCS2,HSD17B7,IDI1,LSS,MSMO1,MVD,MVK,SC5D,SQLE,TM7SF2** |
| **Cholesterol Biosynthesis I** | **7.00** | **0.615** | **-2.828** | **CYP51A1,FDFT1,HSD17B7,LSS,MSMO1,SC5D,SQLE,TM7SF2** |
| **Cholesterol Biosynthesis II (via 24,25-dihydrolanosterol)** | **7.00** | **0.615** | **-2.828** | **CYP51A1,FDFT1,HSD17B7,LSS,MSMO1,SC5D,SQLE,TM7SF2** |
| **Cholesterol Biosynthesis III (via Desmosterol)** | **7.00** | **0.615** | **-2.828** | **CYP51A1,FDFT1,HSD17B7,LSS,MSMO1,SC5D,SQLE,TM7SF2** |
| Hepatic Fibrosis Signaling Pathway | 6.320 | 0.12 | 3.703 | ATF4,BCL2,CACNB2,Calm1 (includes others),CASP3,CCN2,CEBPB,COL3A1,FGFR1,FLT1,FLT4,FOS,FOXO1,FZD4,GLI1,IKBKB,IRS2,ITGA2,ITGA2B,ITGA3,ITGA6,ITGAL,ITGB2,ITGB3,KDR,KLF9,LEF1,LRP1,MAP2K3,MYC,MYD88,MYLK,NFKB2,PDGFB,PDGFRB,PDK1,PIK3R3,PTCH1,PTCH2,RAC2,RAP2B,RND2,SDHD,SNAI1,TFRC,TGFB1,VEGFA,WNT3A,WNT8B,YAP1 |
| tRNA Charging | 6.03 | 0.31 | 3.464 | AARS1,CARS1,EPRS1,GARS1,IARS1,LARS1,MARS1,NARS1,SARS1,TARS1,VARS1,YARS1 |
| **Superpathway of Geranylgeranyldiphosphate Biosynthesis I (via Mevalonate)** | **5.58** | **0.444** | **-2.828** | **ACAT2,FDPS,HMGCR,HMGCS1,HMGCS2,IDI1,MVD,MVK** |
| PTEN Signaling | 5.45 | 0.16 | -2.828 | BCL2,CASP3,CDKN1A,FGFR1,FLT1,FLT4,FOXO1,FOXO3,IKBKB,INPP5B,INPP5D,ITGA2,ITGA2B,ITGA3,ITGA6,ITGAL,ITGB2,ITGB3,KDR,NFKB2,PDGFRB,PIK3R3,RAC2,RAP2B |
| **Mevalonate Pathway I** | **5.37** | **0.5** | **-2.646** | **ACAT2,HMGCR,HMGCS1,HMGCS2,IDI1,MVD,MVK** |
| GP6 Signaling Pathway | 5.08 | 0.17 | 3.441 | Calm1 (includes others),COL13A1,COL3A1,COL4A1,COL4A2,COL5A1,COL6A1,COL7A1,FYB1,FYN,GP6,GRAP2,ITGA2B,ITGB3,LAMB1,LAMB2,LAMC1,LCP2,PIK3R3,RASGRP2,TLN1 |
| Tumor Microenvironment Pathway | 4.59 | 0.14 | 2.858 | BCL2,COL3A1,CXCR4,FGF17,FGF18,FGF19,FGF21,FGF8,FOS,FOXO1,FOXO3,HLA-A,HLA-E,ITGB3,MMP17,MYC,NFKB2,NOS2,PDGFB,PIK3R3,PLAU,RAP2B,SLC1A4,TGFB1,VEGFA |
| ERK/MAPK Signaling | 4.53 | 0.13 | 2.294 | ATF4,CRKL,DUSP6,EIF4EBP1,FOS,FYN,H3-3A/H3-3B,ITGA2,ITGA2B,ITGA3,ITGA6,ITGAL,ITGB2,ITGB3,MKNK1,MYC,MYCN,NFATC1,PAK3,PIK3R3,PPM1L,PPP1R3C,PPP2R5A,PTK2B,RAC2,RAP2B,RAPGEF4,TLN1 |
| Inhibition of Angiogenesis by TSP1 | 4.09 | 0.27 | 2.121 | CASP3,FYN,HSPG2,KDR,NOS3,TGFB1,THBS1,TP53,VEGFA |
| **Zymosterol Biosynthesis** | **3.860** | **0.667** | **-2** | **CYP51A1,HSD17B7,MSMO1,TM7SF2** |

Column 1: Pathway identified by IPA software

Column 2: -log (*p*-value)

Column 3: ratio

Column 4: z-score for pathway

Column 5: Differentially expressed genes within the pathway
