# Supplementary material for: Piezo1 regulates cholesterol biosynthesis to influence neural stem cell fate during brain development
Source: J Gen Physiol. 2022 Sep 7;154(10):e202213084. doi: 10.1085/jgp.202213084 (PMC9458470; doi:10.1085/jgp.202213084)
Supplement: Table S3 — provides a list of antibodies used in this study [file JGP_202213084_TableS3.docx]

**Table S3.** **Antibodies used in this study**

| **Antibody** | **Host** | **Company, Cat #, Conc.** | **Use** | **Dilution** |
| --- | --- | --- | --- | --- |
| anti-ZO-1 | Rabbit | Thermofisher, 61-730-0,  0.25 mg/mL | LSM | 1:50 |
| anti-TUJ1 | Mouse | Santa Cruz, 57670,  0.2 mg/mL | IHC, LSM | 1:200 |
| anti-SOX2 | Rabbit | Millipore Sigma, AB5603,  1 mg/mL | IF, IHC | 1:500 |
| anti-Nestin | Chicken | Abcam, AB150107,  0.3 mg/mL | IHC | 1:500 |
| anti-GFAP | Chicken | Millipore Sigma, AB5541,  50 µL (Lot 3489761 & 3436166) | IF | 1:500 |
| anti-MAP2 | Mouse | Millipore Sigma, M4403,  Ascites fluid (Batch 90707) | IF | 1:200 |
| anti-O4 | Mouse | R&D Systems, MAB1326-SP,  0.1 mg/mL | IF | 1:100 |
| anti-Mouse IgM-555 | Goat | Invitrogen, A21426, 2 mg/mL | IF | 1:500 |
| anti-Chicken IgY-488 | Goat | Fisher, A11039, 2 mg/mL | IHC | 1:500 |
| anti-Mouse IgG-647 | Donkey | Abcam, AB150107, 2 mg/mL | IF, IHC, LSM | 1:500 |
| anti-Rabbit IgG-555 | Goat | Invitrogen, A21428, 2 mg/mL | LSM | 1:500 |

**Light sheet microscopy (LSM), Immunohistochemistry (IHC), Immunofluorescence (IF)**
