# Supplementary material for: Piezo1 regulates cholesterol biosynthesis to influence neural stem cell fate during brain development
Source: J Gen Physiol. 2022 Sep 7;154(10):e202213084. doi: 10.1085/jgp.202213084 (PMC9458470; doi:10.1085/jgp.202213084)
Supplement: Table S4 — lists the microscopes used. [file JGP_202213084_TableS4.docx]

**Table S4. Microscopes**

| **Microscope** | **Objective Magn.** | **Objective Numerical Aperture** | **Image Resolution**  **Pixel Size**  (µm/pixel) | **Camera/Detector** | **Filter cubes**  Fluorescence Excitation/Emission |
| --- | --- | --- | --- | --- | --- |
| Keyence BZ-X810 Widefield Microscope | 10x  20x  60x | 0.45  0.75  1.4 oil | 1.50977  0.75488  0.12581 | 2/3 inch, 2.83 mega pixel monochrome CCD | Ex 340-380/Em 435-485  Ex 465-495/Em 510 and up  Ex 527-553/Em 577-633  Ex 590-65/Em 662-737 |
| Olympus FV3000 Confocal | 40x  60x | 1.25  1.25 | 0.3877  4.8272 | PMT | U-FUW Ex 365/50/Em 420 LP  U-FBN Ex 482/25/Em 510 LP  UFGW Ex 540/20/Em 575 LP |
| Zeiss Z-1 Lightsheet | 5x | 0.16 | 1.3 | s-CMOS camera PCO.edge | 568-40 laser/BP 575-615  638-75 laser/LP 640 |
| Incucyte S3 | 10X | 0.30 | 1.24 | 12 bit CMOS camera | Red 565–605 nm: 625–705 nm |
| Zeiss Elyra 7 | 63x | NA 1.40 Corr  WD: 0.35 | X/Y: 0.031  Z: 0.091 | Dual camera: 2 pco.edge 4.2 camera system with lattice sim leap mode. | 561 TV 1: BP 570-620 + LP 655  488 TV 2: BP 420-480 + BP 495-550  405 TV 2: BP 420-480 + BP 495-550 |
